# Smartphone-Supported versus Full Behavioural Activation for Depression: A Randomised Controlled Trial

**DOI:** 10.1371/journal.pone.0126559

**Published:** 2015-05-26

**Authors:** Kien Hoa Ly, Naira Topooco, Hanna Cederlund, Anna Wallin, Jan Bergström, Olof Molander, Per Carlbring, Gerhard Andersson

**Affiliations:** 1 Department of Behavioural Sciences and Learning, Linköping University, Linköping, Sweden; 2 Department of Psychology, Stockholm University, Stockholm, Sweden; 3 Wemind Psykiatri Stockholm, Stockholm, Sweden; 4 Department of Clinical Neuroscience, Center for Psychiatry Research, Karolinska Institute, Stockholm, Sweden; University of Groningen, Faculty of Social and Behavioral Sciences, NETHERLANDS

## Abstract

**Background:**

There is need for more cost and time effective treatments for depression. This is the first randomised controlled trial in which a blended treatment - including four face-to-face sessions and a smartphone application - was compared against a full behavioural treatment. Hence, the aim of the current paper was to examine whether a blended smartphone treatment was non-inferior to a full behavioural activation treatment for depression.

**Methods:**

This was a randomised controlled non-inferiority trial (NCT01819025) comparing a blended treatment (*n*=46) against a full ten-session treatment (*n*=47) for people suffering from major depression. Primary outcome measure was the BDI-II, that was administered at pre- and post-treatment, as well as six months after the treatment.

**Results:**

Results showed significant improvements in both groups across time on the primary outcome measure (within-group Cohen’s *d*=1.35; CI [−0.82, 3.52] to *d*=1.47; CI [−0.41, 3.35]; between group *d*=−0.13 CI [−2.37, 2.09] and *d*=−0.10 CI [−2.53, 2.33]). At the same time, the blended treatment reduced the therapist time with an average of 47%.

**Conclusions:**

We could not establish whether the blended treatment was non-inferior to a full BA treatment. Nevertheless, this study points to that the blended treatment approach could possibly treat nearly twice as many patients suffering from depression by using a smartphone applica¬tion as add-on. More studies are needed before we can suggest that the blended treatment method is a promising cost-effective alternative to regular face-to-face treatment for depression.

**Trial Registration:**

Cognitive Behavioral Therapy Treatment of Depression With Smartphone Support NCT01819025

## Introduction

There is need for more cost and time effective treatments for major depression [1,2]. One possibility is to use smartphone-delivered psychological treatments [3]. A challenge associated with using mobile phones as platform for psychological treatment is that the user must be able to interact with the program in a fast and easy way [4]. It has been suggested that singular treatment components, such as activity scheduling in behavioural activation (BA), might be a better target for smartphone applications than entire multi-component treatment packages [5]. BA has been found to be effective in a series of meta-analyses in the treatment of depression [6,7], and can be delivered by non-specialists [8]. In addition, recent research has shown that BA can be delivered with the use of new mobile technologies (e.g. smartphones) [5].

In light of the research support for BA [9] as well as of the initial promising findings of smartphone-delivered psychological treatment for depression [3,5], we assumed that a combination of these two aspects might be useful to explore in such a way that the number of treatment sessions would be reduced by means of the smartphone applications. The evaluation of combined treatment methods shows promising results (e.g., combining an internet treatment program with live group sessions for social phobia) [10]. However, these treatment programs mainly rely on self-help exercises, reading texts, and online group discussions with the support from therapist-guided sessions [10]. The starting point of our treatment was traditional face-to-face treatment with the addition of a smartphone tool for support. The goal was to reduce the therapist time in a full face-to-face BA treatment while maintaining the same treatment quality. It has recently been suggested that this kind of blended approach should be further investigated in randomised controlled trials, since initial evaluation show that both patients and therapists expect more benefits than drawbacks from this format [11]. The aim of the present study was to evaluate a *blended treatment* that consists of a smartphone activity scheduling application in addition to a face-to-face treatment for major depression. Since it is known that BA works for major depression [9], the study was designed as a non-inferiority trial. Thus, we used four face-to-face sessions plus the smartphone application and compared this outcome against a full regular ten-session program conducted without the smartphone support. We hypothesised that the new treatment would be non-inferior to the standard treatment and consequently, overcome the drawbacks that previous studies have suggested, namely that adding digital components also increases costs and workload among professionals [11,12,13].

## Methods

### Participants and recruitment

Participants were recruited by self-referral via advertisements in Swedish newspapers. The study was conducted at three clinical settings in Sweden: Stockholm University (Clinic for Psychology) and Wemind Psykiatri (Clinic for Psychiatry) in Stockholm, and Linköping University (Clinic for Psychology) in Linköping. Information about the study was published on a public webpage. The Regional Ethics Board of Linköping, Sweden (Diarienummer: 2012-395-31), approved the study. Written informed consent was obtained from all participants by surface mail before the study started. Except from owning a smartphone, potential participants had to meet the following criterions to be eligible for inclusion:

- at least 18 years of age
- a total score of ≥ 5 on the Patient Health Questionnaire Depression Scale (PHQ-9)
- in the last month, consumed none or a fixed dose of medication for depression and anxiety
- no participation in a similar psychological treatment program, i.e. Cognitive behavioural therapy program (CBT)
- no severe comorbid psychiatric condition, which might interfere with the treatment (e.g., bipolar disorder or schizophrenia assessed during a diagnostic interview, using the Mini-International Neuropsychiatric Interview (MINI) [14])
- no other primary medical problems, which would require other treatments
- no severe alcohol problems according to the Alcohol Use Disorders Identification Test (AUDIT) [15] (below the cut-off value of 8 points)
- no assessed risk of suicide using the MINI, and
- suffering from major depression according to the Diagnostic and Statistical Manual of Mental Disorders (DSM-IV) [16].

**Fig 1:**
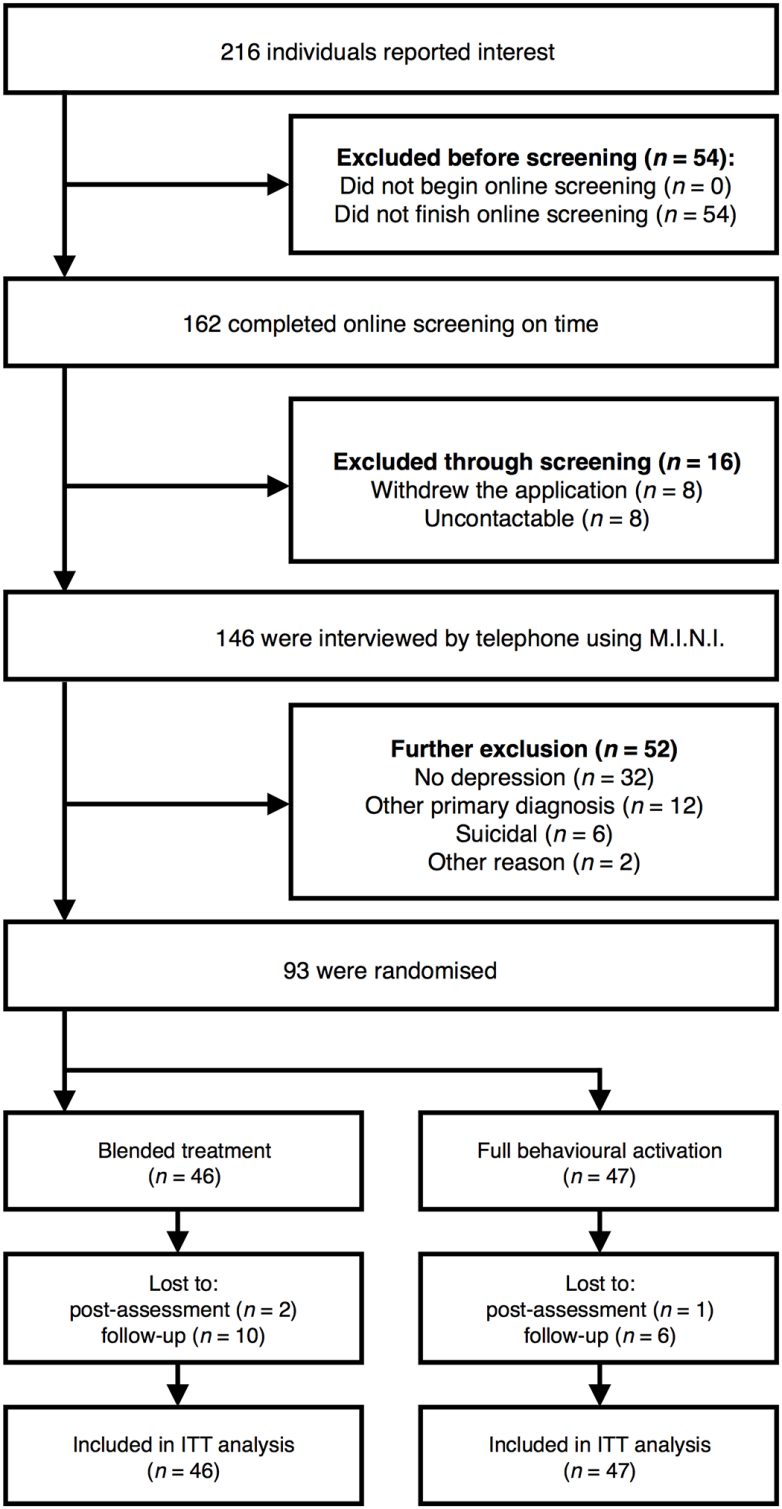
Participant flow and reasons for dropping out throughout the trial.

In the first stage of the recruitment process, potential participants were instructed to complete an online screening survey comprised of the Beck Depression Inventory-II (BDI-II) [17], the PHQ-9 [18], the Beck Anxiety Inventory (BAI) [19], the Quality of Life Inventory (QOLI) [20] the Acceptance and Action Questionnaire (AAQ-II) [21], the Working Alliance Inventory (WAI) [22], the AUDIT [15] and the Drug Use Disorders Identification Test (DUDIT) [23]. Previous psychometric research has validated internet-administration of self-rating scales for depression, quality of life and anxiety [10]. Subsequently, an MSc clinical psychology student conducted a diagnostic assessment phone interview, using the MINI [14] to establish whether the inclusion and exclusion criteria were met. To ensure reliability of the diagnostic procedure, the principal investigator (GA) reviewed all interview protocols together with the assessors. Fig 1 shows the participants’ flow chart throughout the trial and Table 1 informs about the participants’ demographics.

**Table 1:** Demographic description of the participants at randomisation.

		Blended treatment (*N* = 46)	Full BA treatment (*N* = 47)	Total (*N* = 93)
**Age**	Mean (*SD)*	30.2 (11.9)	31.0 (11.0)	30.6 (11.4)
	Min-Max	18–71	20–73	18–73
**Gender**	Female	30 (65.2%)	35 (74.5%)	65 (69.9%)
	Male	16 (34.8%)	12 (25.5%)	28 (30.1%)
**Marital status**	Single	22 (47.8%)	19 (40.4%)	41 (44.0%)
	Married	21 (45.7%)	25 (53.2%)	46 (49.5%)
	Divorced/widow/widower	3 (6.5%)	3 (6.4%)	6 (6.5%)
**Highest educational level**	Nine year compulsory school	1 (2.2%)	1 (2.1%)	2 (2.1%)
	Secondary school	18 (39.1%)	15 (31.9%)	33 (35.5%)
	College/university	27 (58.7%)	31 (66.0%)	58 (62.4%)
**Employment status**	Employed/student	38 (82.6%)	44 (93.6%)	82 (88.2%)
	Unemployed	4 (8.7%)	2 (4.3%)	6 (6.5%)
	Retired	1 (2.2%)	1 (2.1%)	2 (2.1%)
	Other	3 (6.5%)	0 (0.0%)	3 (3.2%)
**Type of Smartphone**	Iphone	23 (50.0%)	22 (46.8%)	45 (48.4%)
	Android	20 (43.5%)	23 (58.9%)	43 (46.2%)
**Medication**	Other	3 (6.5%)	2 (4.3%)	5 (5.4%)
	Yes, earlier	7 (15.2%)	9 (19.1%)	16 (17.2%)
	Yes, present	12 (26.1%)	9 (19.1%)	21 (22.6%)
	None	27 (58.7%)	29 (61.8%)	56 (60.2%)
**Psychological treatment**	Yes, earlier	21 (45.7%)	24 (51.1%)	45 (48.4%)
	Yes, present	2 (4.3%)	2 (4.3%)	4 (4.3%)
	None	23 (50.0%)	21 (44.6%)	44 (47.3%)
**Experience of self-help literature**	Yes	13 (28.3%)	12 (25.5%)	25 (26.9%)
	None	33 (71.7%)	35 (74.5%)	68 (73.1%)
**Diagnosis**	Moderate/severe depression	34 (73.9%)	33 (70.2%)	67 (72.0%)
	Mild depression	12 (26.1%)	14 (29.8%)	26 (28.0%)
	Earlier episodes	39 (84.8%)	38 (80.9%)	77 (82.8%)
	Concurrent anxiety diagnosis	22 (47.8%)	19 (40.4%)	41 (44.1%)

### Outcome measures

#### Primary outcome measure

The primary outcome measure was the BDI-II [17] which was administered three times throughout the study: shortly before treatment (pre-treatment), after treatment (post-treatment), as well as six months after the end of the treatment (follow-up).

#### Secondary outcome measures

The PHQ-9 [18], the BAI [19], the QOLI [20] and the AAQ-II [21] were used as additional measures. All secondary outcome measures were collected at pre-treatment, post-treatment and at follow-up. The PHQ-9 was also administered on a weekly basis during the entire treatment period. Thus, there were three measurements on the BAI, the QOLI and the AAQ-II, and a total of 13 measurements on the PHQ-9.

#### Clinician-administered measures

Psychiatric diagnoses were assessed at pre-treatment, post-treatment and at follow-up by administering the MINI [14]. The MINI is a diagnostic interview that, in contrast to several other diagnostic interviews, is completely structured, making it appropriate for other trained assessors rather than only for experienced psychiatrists [14].

#### Credibility, working alliance and adherence assessment

To measure participants’ perceived treatment credibility, Borkovec and Nau’s Credibility/expectancy scale (C-Scale) [24] was distributed after the first week of treatment. To assess the quality of the working alliance, the client version of the 12-item WAI-S [22] was distributed after the third week of treatment. In addition, WAI-S was also administered at pre-treatment in order to measure the expected working alliance.

It has been suggested that adherence to a therapy manual can be evaluated using a questionnaire that, at the end of every session, asks both the therapist and the client, if different core components of a session were completed [25]. For the current study, we created a short questionnaire consisting of 4–5 questions that, according to the manual, relate to the core elements for each session. The questionnaires were administered in paper format in the end of every session, and were answered with “yes” or “no” by both the therapists and the participants. This was done for both interventions.

### Procedure and design

We used the results from the online screening as pre-treatment assessment for the included study participants. The period from the online screening to the onset of the treatments was as soon as possible, but no longer than 4 weeks. All diagnostic interviews (MINI) were conducted by MSc clinical psychology students mentioned below, who at post-measurement were blind to both participants’ conditions and the treatments they provided. At follow-up treatment, the interviews were conducted by other clinical psychology students, who were also blind to both the participant’s condition and the treatment they provided.

The study was designed as a randomised controlled non-inferiority trial. An external person not involved in the study performed the randomisation. A true random number service (www.random.org) was used to ensure complete randomness. The random sequence was generated after inclusion of participants to ensure that assignment of intervention was concealed from the assessing psychologists and researchers involved in the study. Participants were allocated to the blended treatment or to the full BA treatment by using simple randomisation, which in larger samples can be trusted to generate similar numbers of subjects among groups [26]. The protocol for this trial and supporting CONSORT checklist are available as supporting information; see S1 CONSORT Checklist and S1 Protocol.

Power calculation was based on a non-inferiority trial design. We decided to only regard a standardized mean difference of *d* = 0.50 as a clinically distinct and meaningful difference. For the BDI-II, the subsequent non-inferiority margin was 2.50 points, which corresponds to a standardised mean difference of that magnitude, assuming a standard deviation of 5 BDI-II points. Based on Cohen’s tables [27] we estimated that a sample size of 93 participants would be enough to establish a difference of 2.50 BDI-II points with a two-sided *p*-value of 0.05 and an independent t-test.

### Interventions

#### Blended treatment

**Fig 2:**
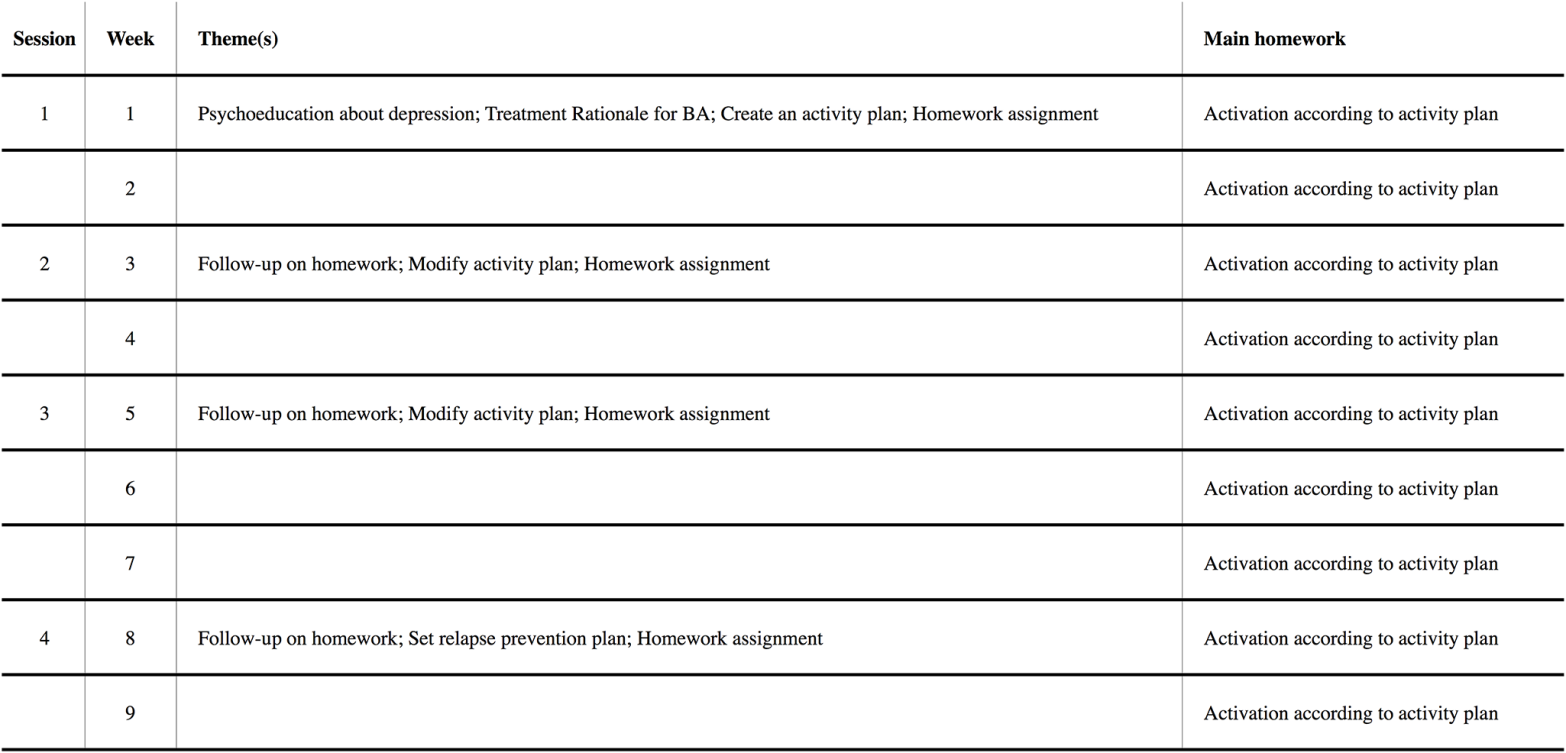
Description of session content for blended treatment.

The blended treatment consisted of four face-to-face sessions and a smartphone application that was used between the sessions. The treatment period ranged over nine weeks with face-to-face sessions every second or third week. The treatment structure was documented in a treatment manual. The manual was created specially for the current intervention by our research group, but inspired by Martell and co-workers’ [9] BA treatment manual. The treatment focused on replacing depressed behaviour with non-depressed (healthy) behaviour that leads to sustainable positive reinforcement. A detailed description of each session can be found in Fig 2. Since we only involved four face-to-face sessions, the current treatment was refined to target the mechanisms of increasing exposure to positive consequences of healthy behaviour and thereby increasing the likely recurrence of such behaviour (see S1 Fig for screenshots of the application). Thus, components such as tackling avoidance and ruminative thinking were not part of the blended treatment. The application was distributed via a web link that was sent out by email prior to the first face-to-face session. The main purpose of the smartphone application was that participants were able to recall and save important non-depressed behaviour to increase everyday activity. This behaviour was specified in consultation with the therapist during the face-to-face sessions. The application also contained a database of 54 non-depressed behaviours divided into three different categories, which participants could add to their application (see SI 2 for the list of behavioural activities in the database). The database aimed to provide suggestions, support, and inspiration for the participants to add their own behavioural activities between the sessions. After a behavioural activity was completed, the participant could save this activity in the application and add a short comment. The participant had access to the statistics and summaries of the quantitative (e.g., behaviour frequency) and qualitative data (e.g., comments). There was also a back-end system, where the therapist could access all participants’ quantitative and qualitative data on a website. The therapist could use the back-end system to send short text messages to the participants via a messaging system, similar to a Short Message Service (SMS). The messaging system was used by the therapists to send personal encouraging messages every other or every third day to the participant as well as weekly general educational messages. The system worked as a one-way communication channel, that is, the participant was not able to respond to the messages. No sensitive personal data, through which the person could be identified, were saved. In addition, all internet (including the therapists’ back-end system) and smartphone activities (including the participants’ mobile application) were secured by means of Secure Sockets Layer (SSL) encrypted information. All participants were using their own smartphones. No monthly service charges for the phone were paid for.

#### Full behavioural activation

**Fig 3:**
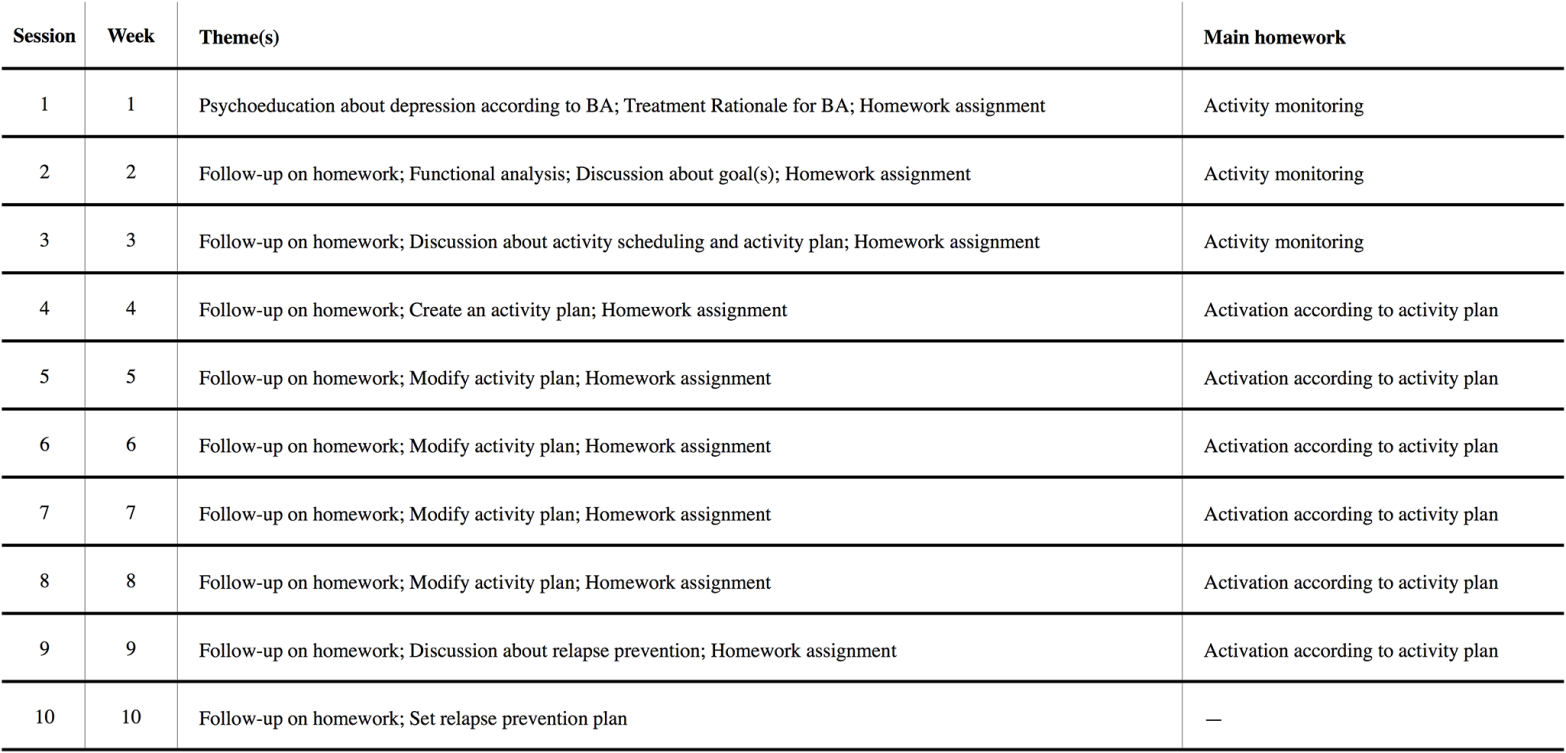
Description of session content for full BA treatment.

Following Martell et al.’s treatment manual, the full comparison treatment consists of a ten face-to-face session BA treatment [28]. This includes the identification of individualized treatment targets, monitoring and scheduling "antidepressant" activities, and reduced avoidance and ruminative thinking. The treatment period ranged over ten weeks with a face-to-face session once a week. Between the sessions, the participants received homework as well as an activity schedule and activity plan in paper format. A detailed description of each session can be found in Fig 3.

#### Therapists

The therapists were final-year students from a five-year M.Sc. clinical psychologist program. All therapists had completed their clinical training. Each therapist was responsible for the treatment of one or two participants of the blended treatment plan and of the full BA. Therapists were randomly assigned to participants with the restriction not to work with more than two participants from each group. Before the treatment started, the therapists were trained in BA, MINI, and the treatment manuals, as well as on how to use the smartphone application and the back-end system. Training sessions were conducted by the principal investigator (GA) in collaboration with the project manager (KHL). During the entire study, the therapists continuously received supervision from psychotherapists (GA, JB and OM) with CBT orientation, who are experienced working with a BA treatment manual.

### Statistical analysis

All analyses were performed using SPSS 20 (SPSS, Inc., Chicago, IL). Tests of baseline differences between conditions were examined using independent samples t-tests for continuous distributed variables and chi-square tests of independence for categorical data. Primary and secondary continuous outcomes were analysed according to the intention-to-treat principle using mixed effects models given their ability to handle missing data [29]. Differences between the blended treatment and the full BA were primarily investigated by modelling interaction effects between group and time. Random intercept models were selected for all measures.

According to the intention-to-treat principle, the models employed made use of all available data and retained all randomised participants. The mixed-effects models were estimated by means of full information maximum likelihood estimation. Maximum likelihood estimation provides unbiased estimates in the presence of missing observations under the less restrictive assumption that data are missing for ignorable non-random reasons [30].

Recovery after treatment was investigated using the BDI-II. To define responders on the BDI-II, we used Jacobson and Truax [31] procedures for calculating reliable and clinically relevant changes of significance to quantify clinical improvement in depressive symptoms on the BDI-II, which is recommended as a standard reporting method for all published research involving psychological interventions. In calculating reliable and clinically relevant criterions for changes of significance, we used data from the BDI-II manual for clinical means, standard deviations and reliability estimates (Cronbach’s alpha), and data from Dozois et al. [32] for the non-clinical means and standard deviations. On the basis of these data, a participant had to improve by ten points or more from pre-treatment to post- and follow-up treatment to show reliable changes (see Jacobson & Truax [31], for details of calculations). In addition, a score of 13 or below post-treatment and follow-up was required to meet criterions for clinically significant changes. This cut-off score was chosen based on the scoring of the BDI-II [17]. Thus, to meet the criterions for recovery, a participant had to improve by ten points or more and score 13 or below. Differences in rates between treatments were analysed by using chi-square tests. Proportions were based on the full intention-to-treat sample: that is, participants who missed data at post- and follow-up treatment were regarded as non-responders.

The non-inferiority margin for the mean difference of change in total depression scores between the treatments for BDI-II was predefined as 2.50 points [33,34]. This margin separate a clinically important treatment difference from a clinically negligible one regarding the outcome measures and have been suggested in earlier non-inferiority trials [33,34]. The confidence interval approach was used to test non-inferiority, that is, if the two-sided 95% confidence limit is completely below the pre-specified positive non-inferiority margin (2.50), the blended treatment would be understood not to be inferior to the full BA. This is equivalent to performing a 2.5% significance test on the null hypothesis, which states that the treatment is inferior by the non-inferiority margin or more as compared to the condition [35]. Effect sizes (Cohen’s *d*) were calculated by dividing the differences in means by the pooled standard deviations [36], based on the intention-to-treat sample.

## Results

The two groups did not differ significantly on any of the measures at pre-treatment (*t* = −0.99 to 0.78, *df* = 91, *p* = .33 to .92). See Table 2 for all outcome measurements at pre-, post- and follow-up treatment.

**Table 2:** Means, SDs and effect sizes (Cohen’s d) for measures of depression, anxiety, psychological flexibility and quality of life.

Mean (*SD*)				Effect size, *d* (95% CI)			
Outcome measure	Pre-treatment	Post-treatment	Follow-up	Between-group, pre to post	Between-group, pre to follow-up	Within-group, pre to post	Within-group, pre to follow-up
BDI-II							
Blended treatment	28.96 (8.07)	15.17 (11.51)	14.61 (12.86)	-0.13 (-2.37, 2.09)	-0.10 (-2.53, 2.33)	1.40 (-0.61, 3.41)	1.35 (-0.82, 3.52)
Full BA treatment	27.32 (7.89)	13.68 (10.68)	13.43 (11.27)			1.47 (-0.41, 3.35)	1.44 (-0.50, 3.39)
PHQ-9							
Blended treatment	15.39 (4.73)	7.13 (5.78)	7.20 (6.13)	0.01 (-1.20, 1.23)	0.05 (-1.18, 1.27)	1.58 (0.51, 2.65)	1.51 (0.41, 2.62)
Full BA treatment	15.30 (4.49)	7.21 (6.27)	7.49 (6.06)			1.50 (0.41, 2.59)	1.48 (0.42, 2.55)
BAI							
Blended treatment	15.74 (12.11)	8.11 (9.27)	12.57 (12.30)	0.36 (-1.48, 2.20)	0.03 (-2.30, 2.37)	0.72 (-1.46, 2.89)	0.26 (-2.20, 2.73)
Full BA treatment	17.47 (9.18)	11.34 (9.02)	12.96 (10.87)			0.68 (-1.14, 2.50)	0.45 (-1.56, 2.47)
AAQ-II							
Blended treatment	30.41 (7.15)	25.78 (7.09)	24.30 (8.37)	-0.01 (-1.62, 1.60)	-0.07 (-1.83, 1.68)	0.66 (-0.78, 2.10)	0.79 (-0.78, 2.37)
Full BA treatment	30.64 (7.68)	25.72 (8.82)	23.66 (9.06)			0.60 (-1.05, 2.26)	0.84 (0.84, 2.52)
QOLI							
Blended treatment	-0.68 (1.72)	0.81 (1.99)	0.82 (2.08)	0.01 (-0.36, 0.37)	0.03 (-0.35, 0.40)	0.80 (0.43, 1.18)	0.79 (0.41, 1.18)
Full BA treatment	-0.44 (1.44)	0.79 (1.65)	0.78 (1.67)			0.80 (0.49, 1.11)	0.79 (0.48, 1.10)

Abbreviations: BDI-II: Beck Depression Inventory-II; PHQ-9: 9-item Patient Health Questionnaire Depression Scale; BAI: Beck Anxiety Inventory; AAQ-II: Acceptance and Action Questionnaire; QOLI: Quality of Life Inventory.

### Attrition and adherence

Out of the 93 randomised participants, five participants (one from blended treatment and four from full BA) decided not to start the treatment. Nevertheless, these were included in the intention-to-treat analysis. Another three (two from blended treatment and one from full BA) participants (totalling 8.6%) did not submit post-treatment data. From the follow-up treatment, another 16 participants, ten from the blended treatment group and six from the full BA group (totalling 25.8%), did not submit data on the self-report measures.

Adherence to treatment was defined as the number of completed face-to-face sessions. Out of 88 participants, who started the treatment, 81 (92%) succeeded to adhere to the entire treatment. 42 (93.3%) participants of those were in the blended treatment group and 39 (92.9%) were in the full BA treatment group.

### Primary outcome measures

**Fig 4:**
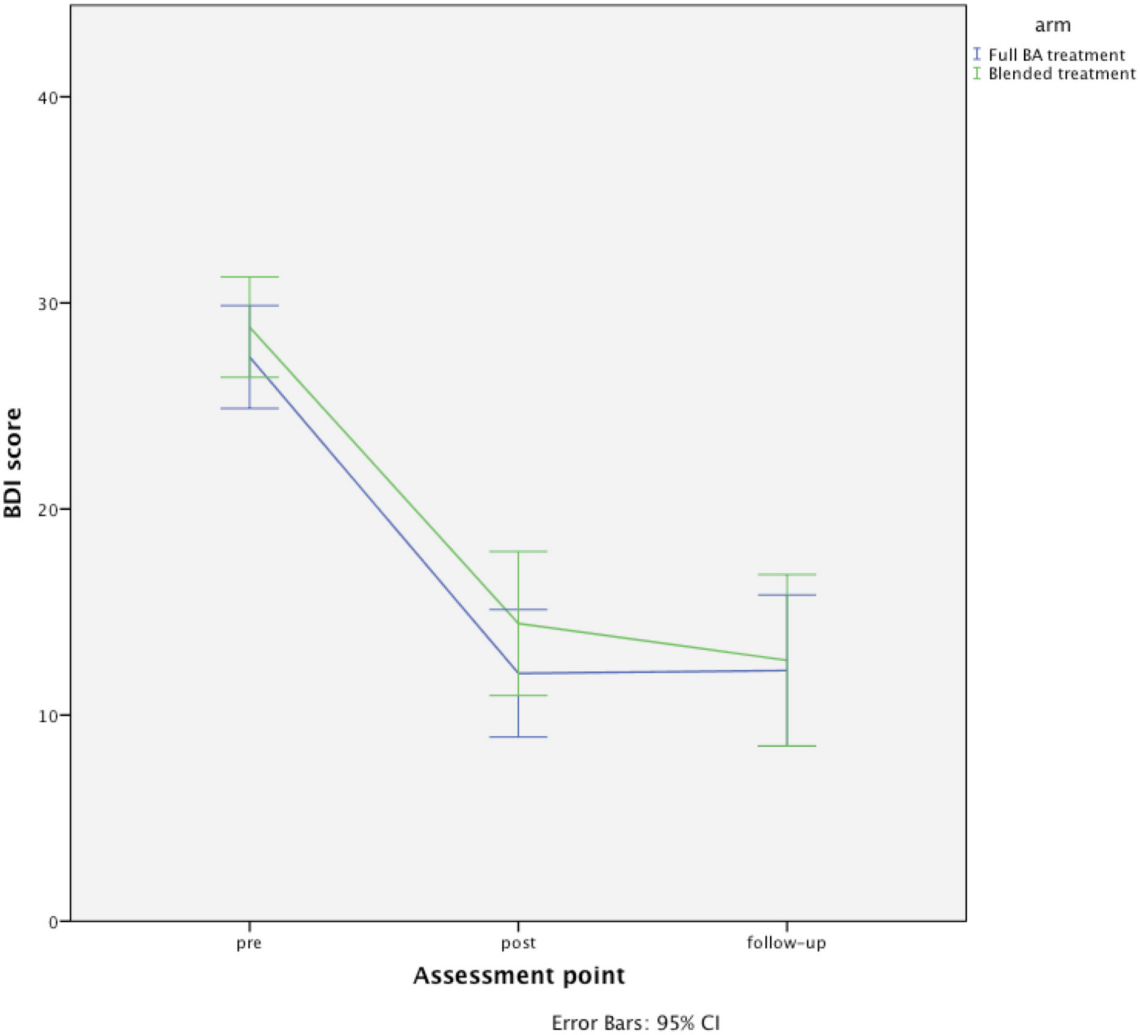
Assessment of the Beck Depression Inventory-II, BDI-II (including 95% confidence intervals).

No significant interaction of group and time was found between groups on the BDI-II, neither between pre- and post-treatment (BDI-II: (*F*_1, 91.13_ = 0.09, *p* = .76)) nor between pre- and follow-up treatment (BDI-II: (*F*_1, 171.81_ = 0.13, *p* = .72)). This is illustrated in Fig 4. The estimated mean difference (95% CI) on the BDI-II between treatments (blended treatment vs. full BA) was 2.42 (CI 95% = −2.19 to 7.03) at post-treatment and 0.50 at follow-up (CI 95% = −4.93 to 5.92). Hence, the upper limit of the 95% CI was not below the pre-specified non-inferiority margin of 2.50 points.

Based on estimated means, the within-group standardized effect sizes for the two treatments were substantial on both post- and follow-up treatment (*d* = 1.35; CI [−0.82, 3.52] to *d* = 1.47; CI [−0.41, 3.35]). The between-group standardized mean differences were absent on both post-treatment (*d* = −0.13 CI [−2.37, 2.09]) and follow-up treatment (*d* = −0.10 CI [−2.53, 2.33]). This is shown in Table 2. The average BDI-II scores for the blended treatment group decreased from 28.96 to 15.17, an average reduction of 13.79 from pre-treatment to post-treatment with an additional decrease of 0.56 points at follow-up. For the full BA group, the decrease was from 27.32 to 13.68, an average reduction of 13.64 and remained stable at follow-up (13.43).

### Secondary outcome measures

No significant interaction effects for group and time were found on the secondary measures neither between pre-treatment to post-treatment (PHQ-9: (*F*_1, 831.27_ = 0.52, *p* = .47); BAI: (*F*_1, 88.80_ = 0.24, *p* = .62); AAQ-II: (*F*_1, 90.20_ = 0.16, *p* = .70); QOLI: (*F*_1, 88.32_ = 0.31, *p* = .58)) nor between pre-treatment to follow-up treatment (PHQ-9: (*F*_1, 911.85_ = 0.11, *p* = .74); BAI: (*F*_1, 162.05_ = 0.34, *p* = .56); AAQ-II: (*F*_1, 166.88_ = 0.01, *p* = .91); QOLI: (*F*_1, 165.17_ = 1.06, *p* = .31)). As shown in Table 2, medium-to-large within-group effect sizes were revealed on all secondary outcome measures (*d* = 0.60; CI [−1.05, 2.26] to *d* = 1.58; CI [0.51, 2.65]) between pre-treatment to post-treatment for both treatment groups, with treatment effects maintained at follow-up treatment. This was the case for both groups. In addition, the between-group standardized mean differences were small to absent in favour for the blended treatment (*d* = −0.07; CI [−1.83, 1.68] to *d* = 0.36 CI [−1.48, 2.20]).

### Recovery rates

As seen in Table 3, there were no significant differences in recovery rates between both treatment groups neither at post-treatment (*χ* ^2^(*N* = 93, *df* = 1) = 0.27, *p* = .60 nor at follow-up treatment (*χ* ^2^(*N* = 93, *df* = 1) = 0.01, *p* = .93. At post-treatment, 21 (45.7%) of the participants of the blended treatment group were classified as responders according to the Jacobson and Truax criterions [31] compared to 24 participants (51.1%) in the full BA group, while at follow-up treatment, 21 participants (45.7%) of the blended treatment group and 21 participants (44.7%) of the full BA group were recovered. In comparison, when recovery was defined as 50% reduction the results at post-treatment showed that 52.2% in the blended treatment group and 59.6% in the full BA group would have been classified as responders. At follow-up, 50.0% in the blended treatment group and 46.8% in the full BA group reported a reduction of 50% or more.

**Table 3:** Proportion of participants reaching recovery (clinically significant improvement on the BDI-II).

	Blended treatment (n)	Blended treatment (%)	Full BA treatment (n)	Full BA treatment (%)	*X* ^2^ Blended vs. Full BA
Recovered post	21	45.7	24	51.1	0.27
Recovered FU	21	45.7	21	44.7	0.01

Abbreviations: BDI-II: Beck Depression Inventory-II; Blended: Blended treatment; Full BA: Full behavioural treatment; Post: Post-treatment; FU: 6 month follow-up.

### Credibility, working alliance and adherence to therapy manuals

Treatment credibility ratings (C-scale) after one week of treatment showed that participants in both groups rated their respective treatment as credible. Out of a possible total of 50, the average scores were 33.0 (*SD* = 8.0) for the blended treatment group and 33.5 (*SD* = 6.5) for the full BA group. Independent t-tests showed no significant differences between both groups at the C-scale (*t*_84_ = −0.30, *p* = 0.76).

The results of the Working Alliance Inventory showed no significant differences between both groups (*t*_91_ = −0.32 to *t*_77_ = 0.89, *p* = .75 to .37). At pre-treatment, the blended treatment group scored 59.0 (*SD* = 11.1), and the full BA group scored 59.7 (*SD* = 10.7); after three weeks, the blended treatment group scored 63.5 (*SD* = 9.6) and the full BA group 65.7 (*SD* = 11.3). When analysing the interventions separately with regards to changes in the outcomes, there were significant correlations on all measurements except from the BAI for the blended treatment group from pre- to post-measurement (*r* = −.56 to .31, *p* = 0.00 to 0.05), but no significant correlations for the full BA group (*r* = −.22 to .19, *p* = 0.19 to 0.44). Thus, this indicates that the quality of working alliance mediated change in outcomes in the blended treatment, but not in the full BA treatment.

In total, 97.1% of the questions regarding adherence to the therapy manuals were answered with “yes” by the therapists and with 97.5% by the participants. For the blended treatment, 97.4% of the questions were answered with “yes” by the therapists and 98.0% by the participants. As for the full BA treatment, the numbers were 96.9% for the therapists and 97.0% for the participants.

### Therapist time

The guideline regarding duration of one face-to-face session for the therapist in the study was 60 minutes; that is, the full BA has an average therapist time of 600 minutes and the blended treatment 240 minutes. With respect to the blended treatment, however, the time in the back-end system needs to be considered as well. The total time a therapist spent in the back-end system on a participant throughout the treatment varied greatly ranging from 24 to 220 minutes (*M* = 80.67, *SD* = 41.56). There was no significant correlation between therapist time spent in the back-end system and changes on the primary outcome measures (*r* = −.05 to .31, *p* = 0.07 to 0.73), neither from pre-measurement to post-measurement nor from pre-measurement to follow-up measurement. When adding the time to the back-end system, the average therapist time for the blended treatment was 321 minutes, which is 47% less compared to the full BA.

## Discussion

### Main findings

To our knowledge, this is the first study with the aim to investigate the efficacy of a blended treatment that consists of a smartphone application as an add-on to face-to-face treatment for major depression. We could not establish whether the blended treatment was non-inferior to a full BA treatment. Nevertheless, the results showed no major differences between the blended treatment (including four face-to-face sessions and a smartphone application) and the full BA (including ten face-to-face sessions) on any of the outcome variables, neither from pre-measurement to post-measurement nor from pre-measurement to follow-up measurement. Moreover, the results revealed significant improvements from pre- to post-treatment on all the outcome measures in both treatment conditions along with large within-group effect sizes and high recovery rates, whereas treatment effects were kept at follow-up treatment. Also these results are comparable to Cuijpers et al’s [37] meta-analysis on face-to-face therapies for major depression, including CBT, BA, psychodynamic therapy and supportive counselling. In this meta-analysis of 92 studies, the overall response rate, defined as 50% reduction on any depression measure, was 48% [37]. Additionally, Cuijpers and co-workers [37] found that the BDI-II scores dropped on average 12.75 points at post-treatment for all studies using CBT (*n* = 14). In the blended treatment, 52.2% of the participants at post-measurement and 50.0% at follow-up reported a reduction of 50% or more on the BDI-II. The average reduction in BDI-II scores for the blended treatment group was slightly lower than the results in the meta-analysis: 13.79 from pre- to post-treatment, and 14.35 from pre-measurement to follow-up.

Thus, the main findings point towards that the blended treatment can possibly be as effective as a full BA treatment. The blended treatment also achieved comparable scores on the treatment credibility and working alliance as the full BA treatment, although the therapist time was reduced by an average of 47%. This should be seen in the light of the findings of dose—response effects, showing that lower doses of psychotherapy have been associated with poorer outcomes [38,39,40]. For example, one study showed that participants suffering from depression who had more than 9 CBT sessions were 2.5 times more likely to have adequate treatment response than those who had 9 or fewer sessions [41]. This allows us to suggest that the blended treatment method could be a promising cost-effective alternative to regular face-to-face treatment for major depression. However, more studies with larger sample sizes are required to establish this conclusion.

### Limitations

This study has several limitations. First, there was no randomisation to an attention-controlled placebo, which raises the issue of misinterpreting regression to the mean as indicated by two effective treatments. Also, a third group given four face-to-face sessions alone might have helped us to demonstrate the effect of the smartphone-application in isolation. However, the purpose was not to determine the effect of the smartphone application, but to examine its comparability to a full BA treatment—a treatment, which previous research has established as effective for adult depression. A second limitation is that we recruited participants by means of advertisements and not within clinic settings. This limits the generalizability of the results, even if previous findings indicate that BA for depression is effective in regular clinical settings with similar outcomes as in studies involving public recruitments [42]. The mean scores of the BDI-II measures at baseline were comparable to previous studies [3,5] and close to the limit of 29, which is proposed for defining severe depression [17]. A third limitation concerns the therapists in the study, who all were trained psychologists and were in the last semester of training of a five-year program. Therefore, it is possible that experienced therapists would have performed even better. However, the therapists were randomly assigned to participants and were equally distributed among the treatment groups. In addition, adherence to therapist manual was measured and the score was equivalent in both groups for both therapists and participants. Also, for the entire duration of the study, the therapists received continuous supervision from an experienced psychotherapist. A fourth limitation refers to the sample size. Non-inferiority trials usually require large samples to detect small and clinically meaningful differences between two active treatments [43]. We did not have the resources to conduct a study with the assumption of a smaller standardized mean difference (e.g., 0.20) and associated confidence intervals. Therefore, we were not able to establish whether the blended treatment is non-inferior to a full BA treatment at the specified alpha-level (2.5%). A much larger sample would have been needed to detect effect differences between both treatment methods and to obtain more reliable effect estimates for both types of treatments.

### Implications and future research

Despite the limitations, this study points towards that therapists administering a blended treatment method could possibly treat nearly twice as many patients with major depression by using the smartphone application as add-on. We assume that the blended treatment format, in which the number of sessions is replaced by the smartphone support application while at the same time the therapist is still highly involved in the treatment, will have implications for the future planning of psychological services.

This study might pave the way for a broad range of trials including the blended treatment format. However, further studies are needed to establish that the blended treatment format is not inferior to a full treatment.

## Supporting Information

S1 CONSORT Checklist CONSORT 2010 checklist of information to include when reporting a randomised trial. (DOC)

S1 Dataset Original dataset from trial. (SAV)

S1 Fig Screenshots of the smartphone application. (PDF)

S2 Fig Behavioural activities listed in the database. (PDF)

S1 Protocol Trials protocol translated to English. (DOC)

S2 Protocol Trials protocol original in Swedish, part 1. (DOC)

S3 Protocol Trials protocol original in Swedish, part 2. (DOC)
