# Supplementary material for: Smartphone-Supported versus Full Behavioural Activation for Depression: A Randomised Controlled Trial
Source: PLoS One. 2015 May 26;10(5):e0126559. doi: 10.1371/journal.pone.0126559 (PMC4444307; doi:10.1371/journal.pone.0126559)

Supplemental Fig. 1 Screenshots of the smartphone application.

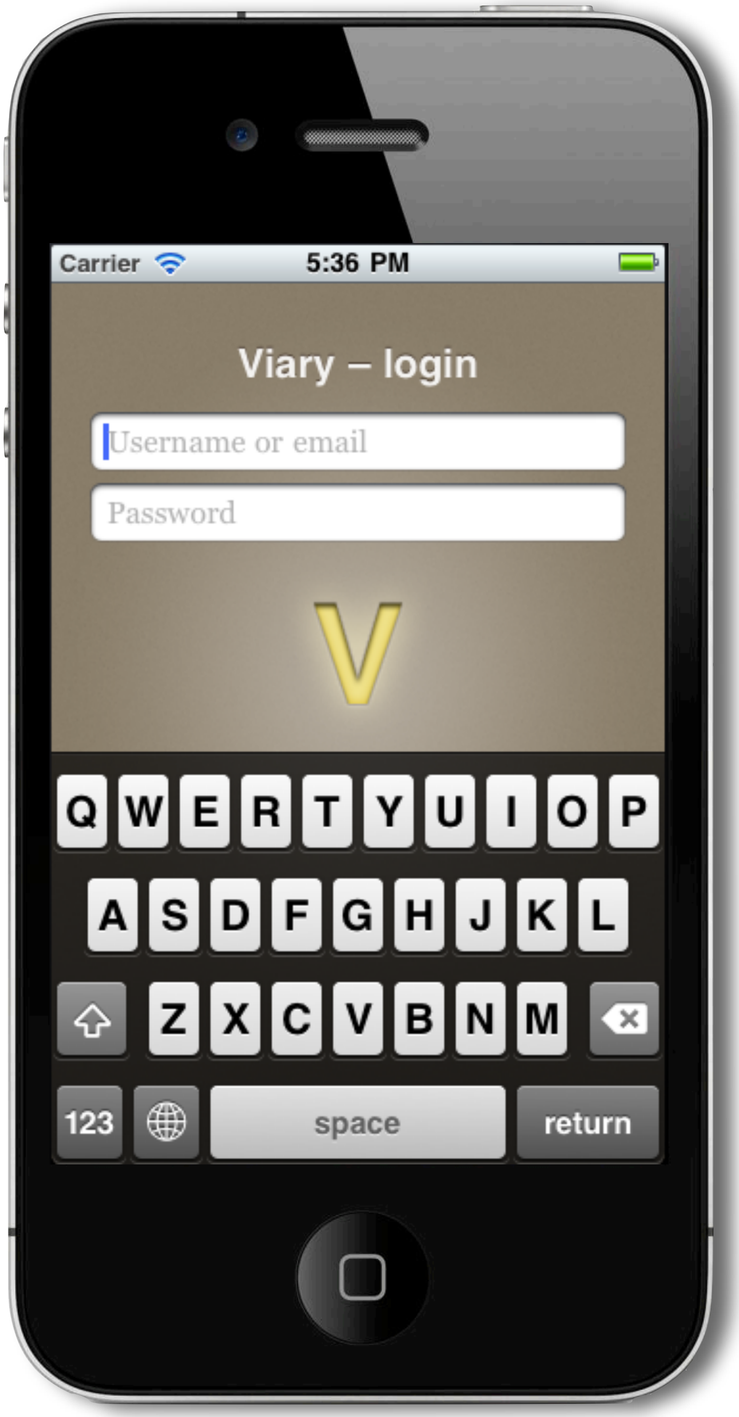

Supplemental Fig. 1 Screenshots of the smartphone application.

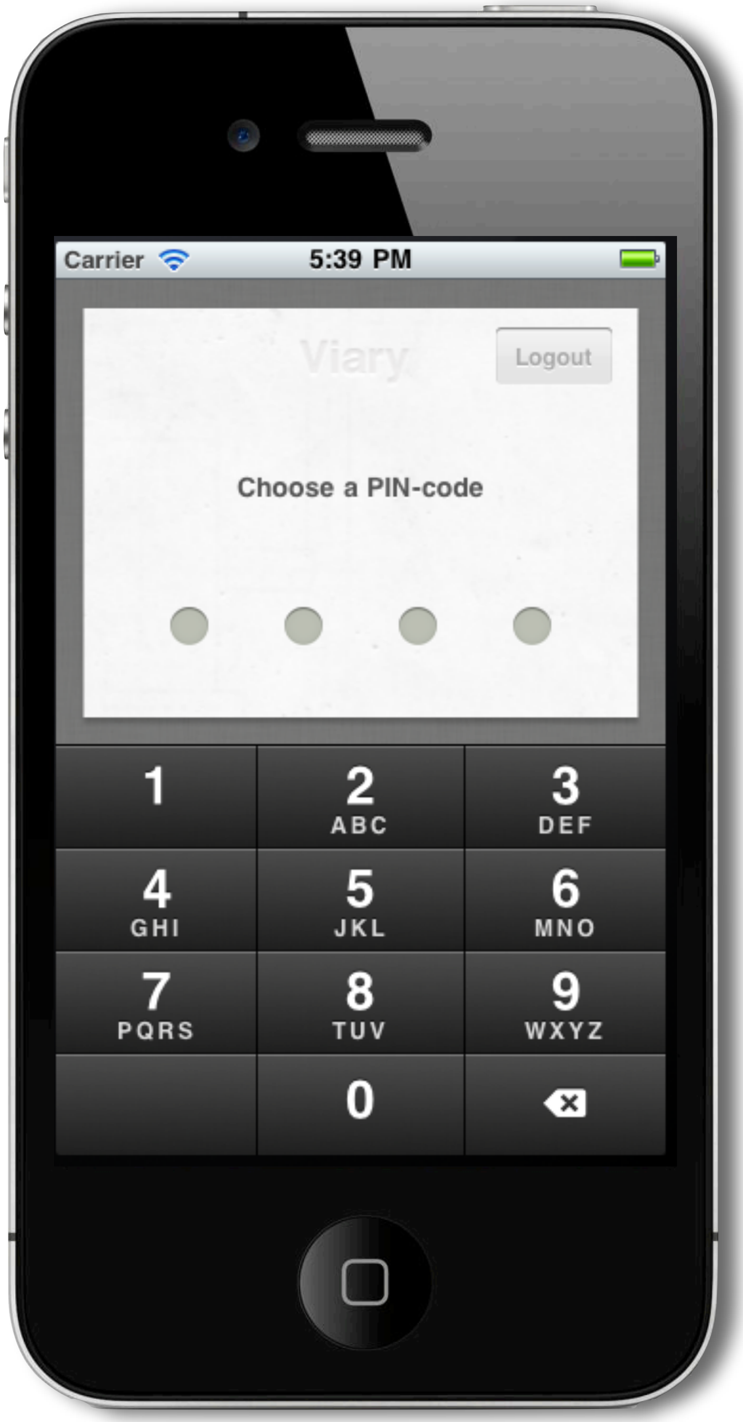

Supplemental Fig. 1 Screenshots of the smartphone application.

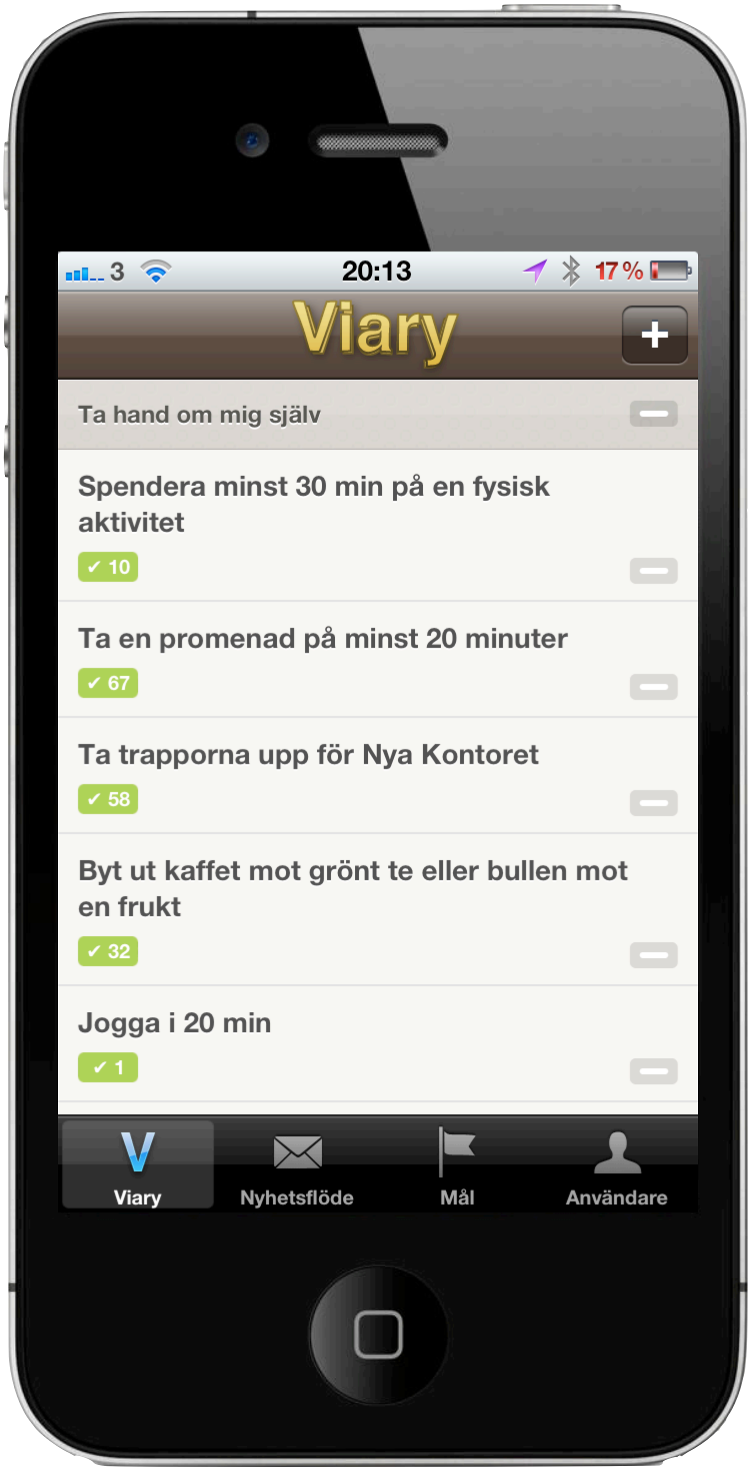

Supplemental Fig. 1 Screenshots of the smartphone application.

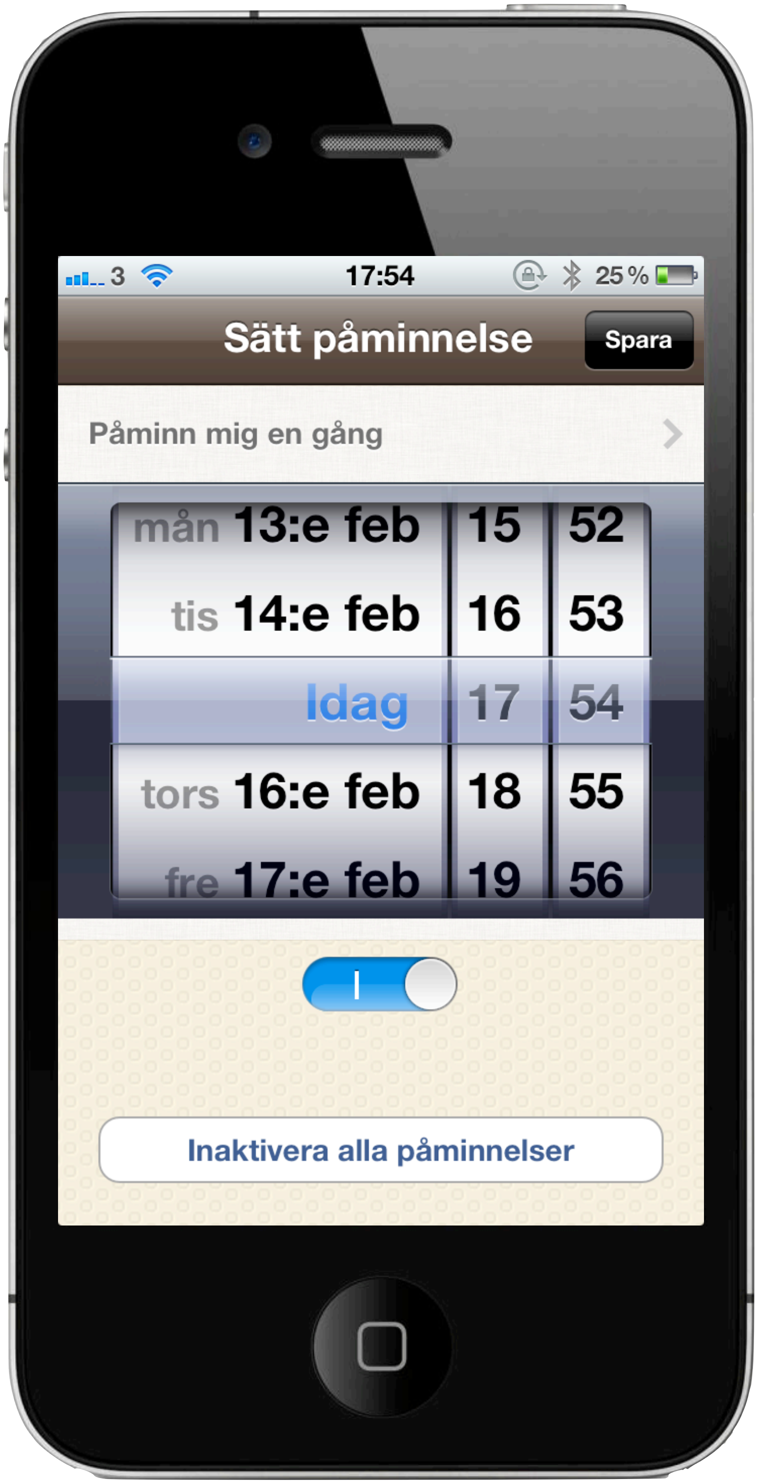

Supplemental Fig. 1 Screenshots of the smartphone application.

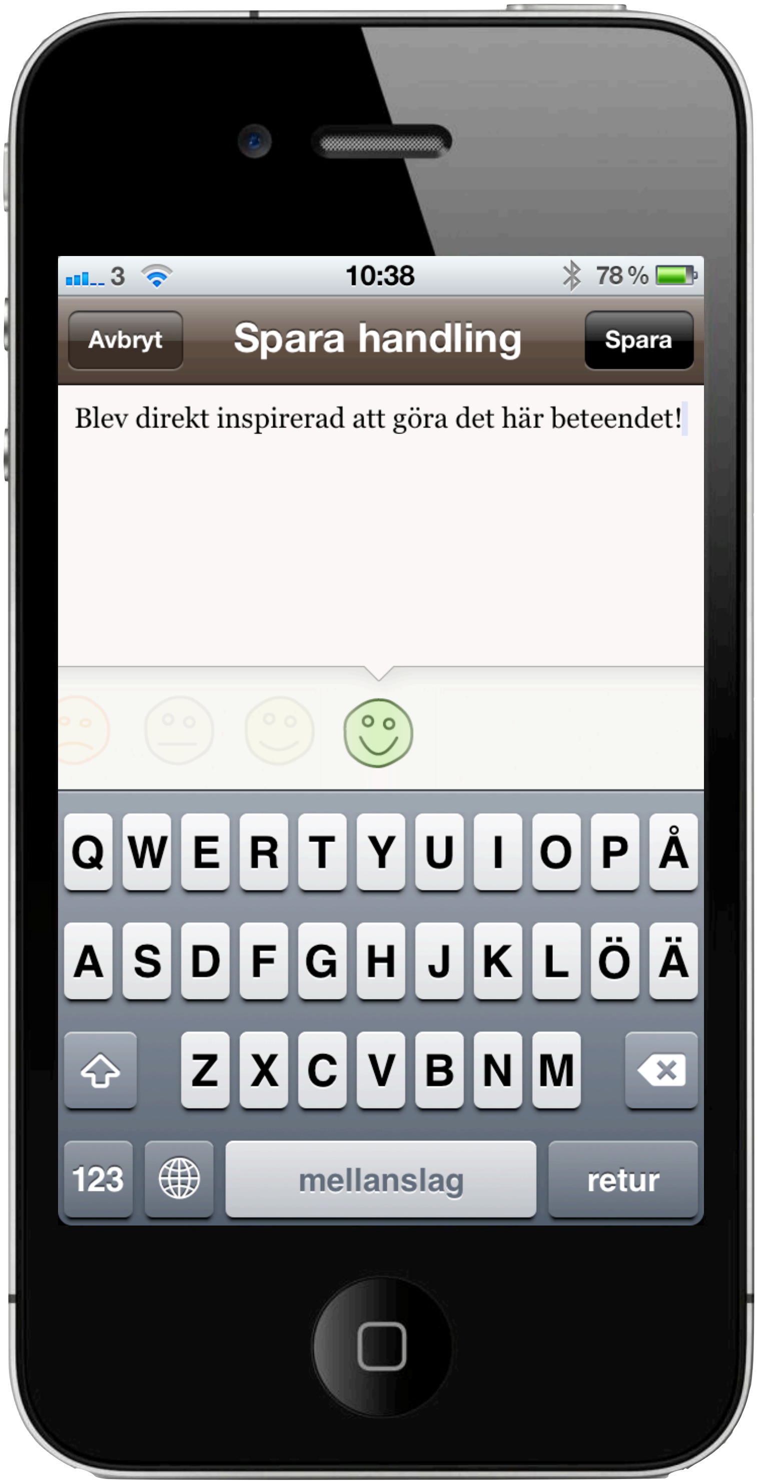

Supplemental Fig. 1 Screenshots of the smartphone application.

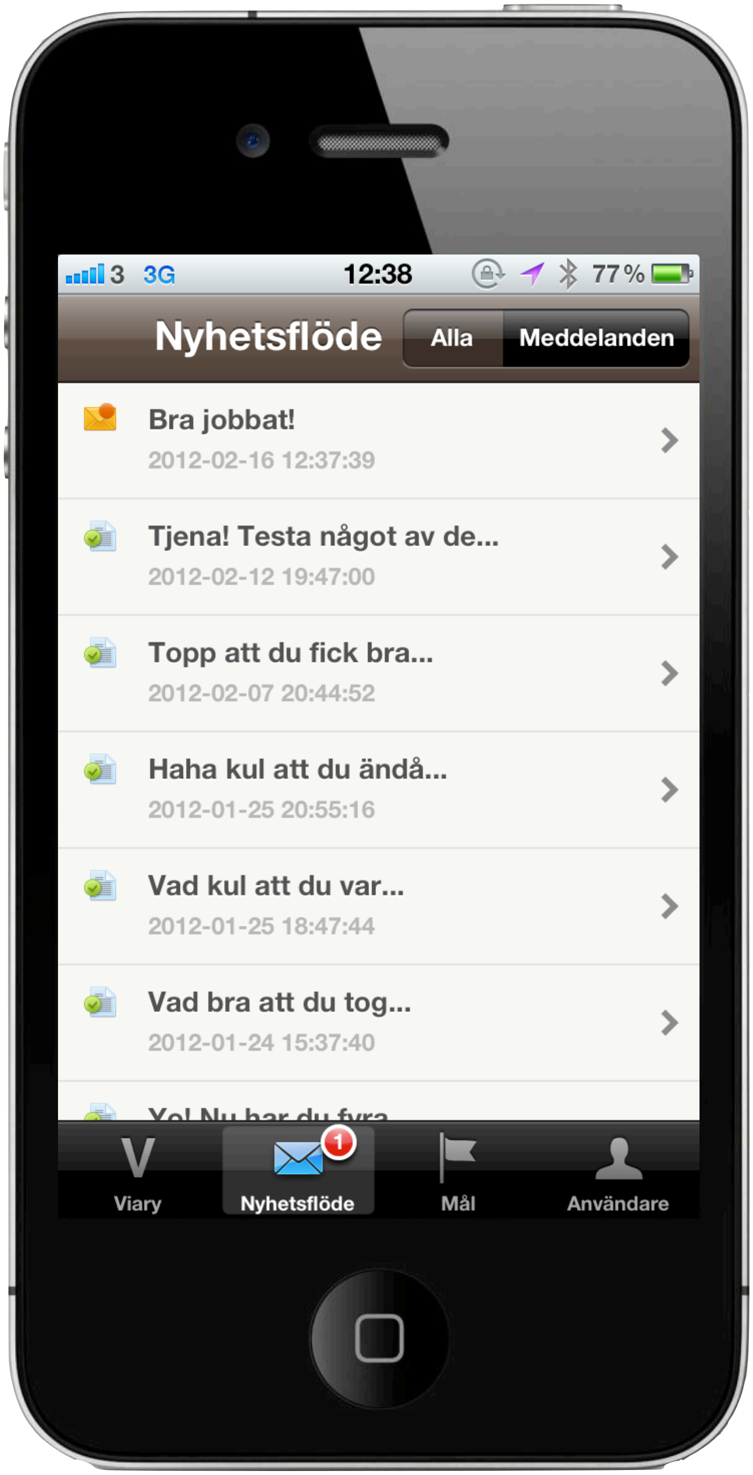

Supplemental Fig. 1 Screenshots of the smartphone application.

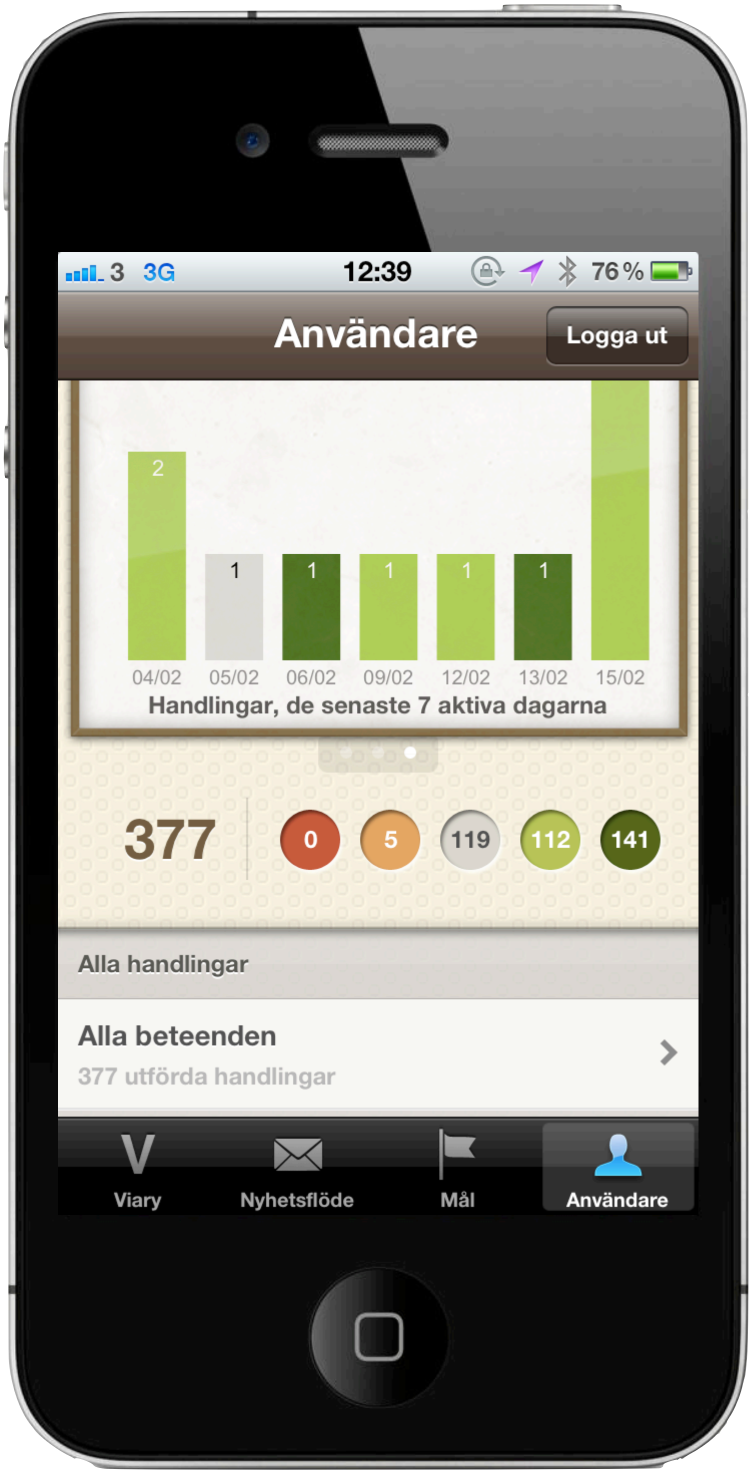

Supplement: S1 Fig — (PDF) [file pone.0126559.s003.pdf]
