# Supplementary material for: Smartphone-Supported versus Full Behavioural Activation for Depression: A Randomised Controlled Trial
Source: PLoS One. 2015 May 26;10(5):e0126559. doi: 10.1371/journal.pone.0126559 (PMC4444307; doi:10.1371/journal.pone.0126559)
Supplement: S2 Fig — (PDF) [file pone.0126559.s004.pdf]

Supplemental Fig. 2 Behavioural activities listed in the database.

Everyday activity structure

Get out of bed when the alarm clock rings in the morning
Taking a shower
Get ready in the morning
Eat breakfast
Read the newspaper
Write a meal plan for each day of the week
Write a shopping list for meals
Buy food for the meals you have planned
Prepare a simple meal
Clean a part of my home
Clean at least 15 minutes
Washing dishes immediately after a meal
Wash my cloths
Plan my TV time with the TV schedule
Turn off the TV before 9 pm, if I'm still watching TV.
Turn off the computer before pm, if I'm still on the Internet
Take a brisk walk for 10 minutes
Log in to my online banking account and pay a bill
Entering my weekly activities in my calendar

Social behaviour

Texting a friend and ask what he / she does
Call a friend and ask what the situation is
Take a walk with a friend
Book a meeting with someone in my family
Suggest a coffee with a friend or family member
Suggest a lunch with a friend or family member
Go to the playground with my kids
Bake something with my children
Meet a friend in the evening and ask how your day was
Watching with a friend an episode of a TV series
Go to the movies with a friend
Cooking with someone

New activities

Buy or borrow a book I wanted to read
Read a book out loud at least 20 minutes
Go to a new cafe and drink coffee
Look up, where the nearest training center is.
Read about online- training
Post a workout plan for the week
Ask a friend if he / she likes to come along and workout
Have at least 30 minutes of physical activity
Listen to a radio program
Watch a documentary on TV
Eat out and have a good meal
Write down at least two good things that happened to me
Rent a movie and have a night in
Look up the exhibits that are in my city
Go to an exhibition at a museum
Look up the concerts that are current right now
Go to a concert
Look up current things happening in my city
Attend a church service
Solve a crossword
Make a Sudoku
Listen to music without doing anything else and focus on what I hear
Go to town and buy something nice for myself
