# Supplementary material for: Smartphone-Supported versus Full Behavioural Activation for Depression: A Randomised Controlled Trial
Source: PLoS One. 2015 May 26;10(5):e0126559. doi: 10.1371/journal.pone.0126559 (PMC4444307; doi:10.1371/journal.pone.0126559)
Supplement: S1 Protocol — (DOC) [file pone.0126559.s005.doc]

**Research Plan**

*Background*

Major depression is expected to be the disorder with the highest disease burden in high-income countries by the year 2030. Estimated costs of depression are annually 177 and 147 million Euro per 1 million inhabitants for major and minor depression respectively. Even if several effective psychological and pharmacological treatments are available for the treatment of depression, it has been shown that current treatment methods are only capable of reducing the burden of disease of depressive disorders with about one third. Therefore, there is a need for cost-effective interventions that can be made available to a larger part of the population suffering from major depressive disorders. Furthermore, behavioural activation is an intervention that can largely benefit from the use of new mobile technologies (e.g. smartphones).

The efficacy of behavioural activation in the treatment of major depressive disorders has been established in a number of studies over the last four decades, but in particular a dismantling study by the late Jacobson showed that behavioural activation was as effective as the full CBT treatment package. In a later study behavioural activation was found to be as effective as antidepressant medication, and more effective than cognitive therapy for the more severely depressed patients. A series of reviews and meta-analyses also show that behavioural activation is at least as effective as full CBT packages that include both cognitive and behavioural components. There are however somewhat different conceptualisations of behavioural activation, with the most recent covering behavioural avoidance, but more less all having activity scheduling as a treatment component based on the early work by Lewinsohn.

*The use of mobile phones in psychological treatment*

Boschen and Casey summarized the advantages of using mobile phones in CBT, and we believe the same advantages apply to smartphones: 1) mobile, 2) accepted in society in general 3) relatively cheap (this also applies to smartphones which on average tend to be less expensive than computers) 4) they are a device with comparatively low ongoing maintenance costs, 5) they are a device already owned by a large number of people, 6) they are almost always on in that they continue to operate, 7) almost always connected 8) they are programmable, meaning they are able to run novel applications software, 9) they are capable of recording media, including audio, photographs, and in many cases video, as well as being able to play or show these media to the user, 10) they are capable of interacting with the user to allow input of data using a keypad, keyboard, or touch screen 11) and they are generally designed to be easy to use for most of the population. Moreover, smartphones can be integrated into CBT homework as their use attracts no attention, allowing users to interact with a handset without fear of stigma or judgment.

One important feature of mobile technology is the possibility for the therapist to reach the patient between sessions and thus create direct incentives for behavioural activation in everyday life. Therefore, developing smartphone-based behavioural activation interventions might be a way to develop a cost-effective treatment for people suffering from major depressive disorders.

*Purpose and potential benefits of the study*

The aim of the current study will be to conduct a randomized controlled trial in which the smartphone depression application will be tested as an adjunct to face-to-face treatment for mild to moderate depression. Since it is known that a full behavioural activation package works for mild to moderate depression, the study will be designed as a non-inferiority trial with fewer face-to-face sessions. Instead of the regular 10-15 session protocols we will include four live sessions and the mobile application as an adjunct to those sessions. As a control condition we will provide full behavioural activation for 10 sessions. Even if we decrease the number of sessions it is still likely that there will be minor differences between the groups. However, we will not only investigate symptoms of depression but also compliance and retention (what is learned from the therapy), and we hypothesize that the smartphone supported behavioural activation treatment will lead to equal compliance and potentially more retention.

A prototype of our smartphone application, together with a psychoeducation, has been tested in a small exploratory study. This prototype was however not directed at depression, and was used as a self-help tool. The results from the pilot study gave us useful information and we concluded that the intervention include some important features that could be further developed and tested. The results also showed that the smartphone application was used by some participants in situations where it was difficult to use a computer connected to the Internet, for example in the underground train, on the bus and train etc. Comments made by two subjects point to another advantage of using mobile phones as platform for health interventions, which has not been highlighted in research before: “just seeing the icon on the phone made me remember things I wanted to accomplish” and “carrying around it is like carrying around your values”. More generally, this might carry implications that the platform of mobile phones does not only assimilate an intervention into a user’s daily life, but also increases the awareness of the intervention, and thus can increase adherence to the intervention. In light of these observations and the literature we believe it is feasible to precede and test if behavioural activation can be presented in the format of a smartphone application.

From a theoretical point of view the trial is important as it tests the possibility of integrating modern information technology with face-to-face therapy. Should the two treatments yield equivalent outcomes it will be informative regarding the use of briefer treatments (from the therapist point of view), which may have implications from a societal perspective.

*Patient Selection and Procedure*

The study will be advertised on the internet and through newspapers. Only persons who already use smartphones (i.e., Iphone, Android) will be invited to participate. After online screening we will interview the potential study participants via telephone using the Structured Clinical Interview for DSM-IV; SCID, which is based on Diagnostic and Statistical Manual of Mental Disorders (DSM-IV). The interview will follow the screening information obtained online and will cover major depression and dysthymia, including number of previous episodes. Separate questions will be asked regarding experience of handling smartphones. Interviews will be conducted by psychologists, and all interviews will be checked by a psychiatrist and careful treatment history will be included (i.e., medication and previous psychotherapy). The primary diagnosis should be major depression of mild to moderate character. Medication will be allowed with the requirement that the dose is stable. Persons with suicidal intent and patients for whom the depression is directly linked to another major primary psychiatric disorder (e.g., psychosis or bipolar disorder) will be excluded and referred to other treatment resources. Included patients will be randomised to two conditions:

1. Four supportive sessions of live CBT for depression and the smartphone application to work with in between the sessions.
2. A full 10 session behavioural activation treatment using the treatment manual by Martell and co-workers.

*Timetable and Evaluation*

Preparations will be made during autumn 2012. This means the revision of manuals for self-help programs, and training of therapists. A website will also be created and then used during the study. People who have registered for the study and screened will be interviewed, later in the period February 2013. After randomisation to experimental group and control group, treatment begins which is expected to last for eight and ten weeks respectively. After stopping treatment, the data will be processed and compiled.

*Project Group*

Gerhard Andersson, Professor, lic. psychologist, Department of Behavioural Sciences and Learning, Linköping University

Kien Hoa Ly, PhD student, Department of Behavioural Sciences and Learning, Linköping University

Naira Topooco, psychologist candidate, Department of Clinical Neuroscience, Center for Psychiatry Research, Karolinska Institute

Christian Ruck, specialist in psychiatry, Stockholm

Thomas Eriksson, specialist in psychiatry, Linköping

References

Andersson, G., & Cuijpers, P. (2009). Internet-based and other computerized psychological treatments for adult depression: A meta-analysis. Cognitive Behaviour Therapy, 38, 196-205.

Andrews, G., Issakidis, C., Sanderson, K., Corry, J., & Lapsley, H. (2004). Utilising survey data to inform public policy: comparison of the cost-effectiveness of treatment of ten mental disorders. The British Journal of Psychiatry, Jun; 184: 526-533.

Andrews, G., Cuijpers, P., Craske MG, McEvoy, P. & Titov, N. (2010). Computer therapy for the anxiety and depressive disorders is Effective, acceptable and practical health care: A meta-analysis, PLoS ONE 5th

Boschen, M. J., & Casey, L. M. (2008). The use of mobile telephones as adjuncts to cognitive behavioral psychotherapy. Professional Psychology: Research and Practice. 39: p. 546-552.

Dimidjian, S., Barrera, M., Martel, C., Muñoz RF & Lewinsohn, PM (2011). The Origins and Current Status of Behavioral activation treatments for depression. Annu Rev Clin Psych, 7, 1-38

Glück, T. M., & Maercker, A. (2011). A randomized controlled pilot study of a breif web-based mindfulness training. BMC Psychiatry, 11, 1-12.

Harrison, V., Proudfoot, J., Wee, PP, Parker, G., Pavlovic, DH, & Mani Cava Sagar, V. (2011). Mobile Mental Health: Review of the emerging field and proof of concept study. Journal of Mental Health, 20, 509-524.

Helbig, S., & Fehm, L. (2004). Problems with homework in CBT: Rare exception or rather frequent? Behavioural and Cognitive Psychotherapy, 32, 291-301

Ly, K. H., Carlbring, P., & Andersson, G. (2012). Behavioral activation-based guided self-help treatment Administered through a smartphone application: study protocol for a randomized controlled trial. Trials.13: 62.

Ly, KH, Dahl, J., Carlbring, P., & Andersson, G. (2012). Development and initial evaluation of a smartphone application based on Acceptance and Commitment Therapy. Springer Plus. 1:11.

Morris, ME, Kathawala, Q., Leen, TK, Gorenstein, EE, Guilak, F., Labhard, M., et al. (2010). Mobile therapy: Case study evaluations of a cell phone application for emotional self-awareness. Journal of Medical Internet Research, 12, e10.

Newman, MG, Szkodny, LE, Llera, SJ & Przeworski, A. (2011). A review of technology-assisted self-help and minimal contact therapies for anxiety and depression: is human contact Necessary for therapeutic efficacy? Clinical Psychology Review, 31, 89-103.

Proudfoot, J., Klein, B., Andersson, G., Carlbring, P., Kyrios, M., et al. (2010). Guided CBT Internet interventions: Specific Issues in Supporting clients with depression, anxiety and co-morbid conditions. In J. Bennett-Levy et al. (Eds.). The Oxford Guide to Low Intensity CBT interventions (pp. 253-264). Oxford: Oxford University Press.

SBU. (2007). Computer-based cognitive behavioral therapy for anxiety disorders or depression. Stockholm: Swedish Council on Technology Assessment in Health Care (SBU).

Sobocki. P. (2006). Health economics of depression (thesis). Stockholm: Karolinska Institutet. Department of Learning, Informatics, Management and Ethics. Medical Management Centre.

Sobocki, P., Jönsson, B., Angst J., & Rehnberg, C. (2006). Cost of depression in Europe. Journal of Mental Health Policy and Economic, 9, 87-98.

Sobocki, P., Lekander, I., Borgstrom, F., Power, O. & Runeson, B. (2007). The economic burden of depression in Sweden from 1997 to 2005. European Psychiatry, 22, 146-152

National Board. (2010). National guidelines for treatment of depression and anxiety disorders - support for control and management. Stockholm: National Board.
