# Supplementary material for: Smartphone-Supported versus Full Behavioural Activation for Depression: A Randomised Controlled Trial
Source: PLoS One. 2015 May 26;10(5):e0126559. doi: 10.1371/journal.pone.0126559 (PMC4444307; doi:10.1371/journal.pone.0126559)
Supplement: S2 Protocol — (DOC) [file pone.0126559.s006.doc]

**Forskningsplan**

*Bakgrund*

Vårdbehovet för psykologisk behandling för personer med depression är större än vad resurserna i hälso- och sjukvården idag kan erbjuda (Socialstyrelsen, 2010). År 2005 led 21 miljoner personer av depression i Europa och det kostar det europeiska samhället över 1000 miljarder SEK årligen (Sobocki, Jönsson, Angst & Rehnberg, 2006). Depression medför samhällskostnader i form av nedsatt produktivitet, ökad sjuklighet, ökad dödlighet samt ökad konsumtion av hälso- och sjukvård (Socialstyrelsen, 2010). I Sverige svarar depression och andra psykiska sjukdomar för en femtedel av den samlade sjukdomsbördan (Sobocki, 2006). Med sjukdomsbörda menas en kombination av antal förlorade friska år genom funktionsnedsättning och antalet förlorade år genom en för tidig död. Den totala kostnaden för depression i Sverige har fördubblats mellan år 1997 och 2005 och år 2005 kostade sjukdomen 32,9 miljarder kronor årligen (Sobocki, Lekander, Borgström, Ström & Runeson, 2007). Siffrorna beräknas både utifrån direkta kostnader såsom vårdkonsumtion och indirekta kostnader i form av sjukskrivningar och därmed förlorat produktionsvärde. Sannolikt är dessa siffror högre idag, då psykisk ohälsa anses vara ett växande problem. Ett exempel på denna utveckling är det ökade procentuella antalet personer som går långtidssjukskrivna eller har aktivitetsbidrag/förtidspensioneras (SBU, 2007).

För att behandla depression har kognitiv beteendeterapi (KBT) visat sig vara verksamt. I SBU:s rekommendationer för behandling av depression hos vuxna, framhålls att effekten av KBT-behandling har starkt vetenskapligt underlag. Även psykofarmaka har visat på god effekt vid mätning direkt efter avslutad behandling. Dock är effekterna av psykofarmaka ofta kortvariga medan behandlingseffekterna av KBT är mer bestående och därmed har en förlängd skyddande effekt som läkemedelsbehandling saknar. Då läkemedel har en rad kända biverkningar, samt att långtidseffekten av de moderna antidepressiva läkemedlen ännu inte är helt kända, är KBT ett bra behandlingsalternativ. Detta motiveras vidare av Socialstyrelsens riktlinjer gällande KBT som förstahandsval vid behandling av mild till måttlig depression.

Även om traditionell KBT generellt har mycket gott stöd på gruppnivå så finns det patienter som inte fullföljer behandling och/eller inte rapporterar symtomlindring. Det finns ett stort antal patienter som avbryter behandlingen i förtid och följsamhet till obligatoriska hemuppgifter är ett stort problem (Helbig & Fehm, 2004). Dessa faktorer reducerar ofta behandlingsframgång och ökar risken för återfall. Således finns ett utvecklingsbehov gällande traditionell KBT-behandling. Man räknar t ex med att nuvarande metoder endast klarar av att minska sjukdomsbördan av depressiva sjukdomar med omkring en tredjedel (Andrews et al., 2004).

För att öka tillgängligheten av psykologisk behandling har en utveckling gått mot mer kostnads- och tidseffektiva behandlingsformat vid sidan av traditionell live-terapi (Newman, Szkodny, Llera & Przeworski, 2011). Förutom självhjälpslitteratur, även kallad biblioterapi, har en stor utveckling skett mot digitalt administrerad terapi, som t ex internetadministrerade behandlingar och behandling via Smartphones. Under de senaste 10 åren har flertalet datoradministrerade psykologiska behandlingar utvecklats för bland annat depression, stress, bipolär sjukdom, missbruksproblematik och ätstörningar (Harrison et al., 2011). Metaanalyser visar att internetadministrerade KBT-behandling ger måttliga till stora effekter på depressionssymptom (Andrews et al., 2010; Andersson & Cuijipers, 2009; Glück & Maercker, 2011). Forskningsfältet kring mobiltelefoni och psykisk hälsa är nytt, men flertalet studier pågår för att utveckla området (Proudfoot et al., 2010). Morris et al. (2010) sammanfattar att studierna är lovande och visar på möjligheten att leverera psykoterapi på ett nytt och effektivt sätt. Boschen och Casey (2008) sammanfattar fördelarna av att använda mobiltelefoner i KBT-behandling med dessa punkter: 1) mobila, 2) accepterade i samhället, 3) relativt billiga, 4) enhet med jämförelsevis låg driftskostnad, 5) enhet som redan ägs av ett stort antal personer, 6) i stort sett alltid påslagna, och därför alltid igång, 7) nästan alltid uppkopplade, 8) programmerbara, vilket betyder att nya program och applikationer kan läggas till, 9) kapabla att spela in ljud, bild och video, samt kunna spela upp dessa olika typer av media, 10) kapabla att interagera med användaren och därmed samla data, och 11) användarvänliga. Dessutom drar användning av mobiltelefon ingen uppmärksamhet till användaren, vilket gör att det är lätt att interagera med enheten utan att behöva känna sig stigmatiserad eller utdömd.

Stor erfarenhet av internetadministrerade KBT-behandlingar finns i forskargruppen. Nyligen gjordes också en stor randomiserad kontrollerad studie med en kombination av internetadministrerad behandling och smartphone-stöd för behandling av depression i forskargruppen. Denna studie blev tidigare godkänd av etikprövningsnämnden, dnr 2011/395-31. I studien utvärderades ett beteendeaktiveringsprogram i en åtta veckor lång vägledd självhjälpsbehandling för personer med subklinisk och egentlig depression. Behandlingen jämfördes mot en aktiv kontrollgrupp baserad på ett mindfulnessprogram. Resultaten visade att båda behandlingarna gav god effekt. För beteendeaktiveringsgruppen d = 1.94 och för mindfulnessgruppen d = 1.19, mätt med huvudutfallsmåttet BDI-II. Signifikanta interaktionseffekter av behandling och tid uppvisades då de deltagarna som tillhörde den halvan som var mest deprimerade (PHQ-9 > 13) analyserades för sig: F(1, 34) = 4,478, p < .05. Effektstorleken för mellangruppsskillnaden var d = .78. Efter behandlingen uppfyllde 73.5 % av deltagarna i beteendeaktiveringsgruppen inte längre diagnosen egentlig depression, enligt M.I.N.I. För mindfulnessgruppen var den siffran 53.1 %. Studieprotokollet publicerades i tidskriften Trials Journal tidigare i våras (Ly et al., 2012a), och vi har precis påbörjat en fyramånadersuppföljning för att se om resultaten håller i sig över tid. Nyligen publicerades också en case study på prototypen till beteendeaktiveringsprogrammet i Springer Plus (Ly et al., 2012b).

Mot bakgrund av det goda forskningsstödet för traditionell KBT och digitalt administrerad KBT vid depression dras slutsatsen att en kombination av dessa behandlingar bör undersökas. Traditionell live-terapi vid depression, särskilt beteendeaktivering, antas kunna dra stor nytta av modern kommunikationsteknik, framför allt mobilteknik, för att göra behandlingen mer effektiv och till en integrerad del i klientens vardag. Beteendeaktivering som en behandlingsform för depression utgår från schemaläggning och hemuppgifter av aktiviteter. Ett särskilt program på mobiltelefonen, så kallad applikation, som fyller denna funktion förväntas kunna vara ett stöd i traditionell live-terapi som gör denna behandling mer effektiv. Denna applikation användes och testades i ovan nämnda studie.

*Syfte och nytta med studien*

Syftet med studien är att är att undersöka om live-terapi tillsammans med en smartphone-applikation, inriktad på att ge ett stöd i hemuppgifter samt en ökning av beteendeaktivering, är effektiv i behandling av mild till måttlig depression. Studien kommer att genomföras som en randomiserad kontrollerad behandlingsstudie där effekten av behandlingen jämförs med en kontrollgrupp. Eftersom det har visat sig att fullständig beteendeaktivering är en effektiv behandling av mild till måttlig depression (Dimidjian et al., 2011), kommer studien utformas som en så kallad non-inferiority-studie där behandlingsgruppen ges färre möten live, men istället stöd i form av smartphone-applikationen. Istället för en traditionell beteendeaktiverings-behandling på 10 sessioner, kommer behandlingsgruppen att ges fyra live-terapi sessioner samt smartphone-applikationen, som ett komplement och stöd till de fyra sessionerna. Som kontrollgrupp kommer vi att ge full beteendeaktivering med 10 sessioner, i live-terapi. Även om vi minskar antalet sessioner i behandlingsgruppen är hypotesen att det fortfarande kommer att finnas små skillnader mellan grupperna vad gäller behandlingseffekt och symptom. Studien kommer dock inte bara undersöka effekt på depression, utan också följsamhet i behandling och vidmakthållande av effekt samt symptom. Hypotesen är att den kombinerade behandlingen av live-terapi och smartphone-stöd kommer att leda till lika stor följsamhet och potentiellt större vidmakthållande effekter än kontrollgruppen.

Studien utgår från befintliga utforskade behandlingsmodeller inom kognitiv beteendeterapi (KBT), nämligen beteendeaktivering (Dimidjian et al., 2011). Innehåll i den föreliggande studiens behandlingar kommer också nyttja erfarenheter och innehåll från det flertal internetbaserade psykologiska behandlingar som tidigare utforskats av forskarlaget, samt den nyligen gjorda studien med smartphone-stöd. Om behandlingen skulle visa sig ha lika god effekt som kontrollgruppen skulle detta innebära ett starkt komplement till traditionell psykologisk behandling och psykofarmakologisk vård som erbjuds patienter med mild till måttlig depression. Om det visar sig att tekniskt stöd påverkar psykologisk behandling positivt så uppstår en mängd utvecklingsmöjligheter. Det kan på sikt leda till positiva konsekvenser såsom ökad tillgänglighet, minskade vårdköer samt effektivare behandling avseende kostnader och behandlingsutfall.

*Patienturval och procedur*

Deltagarna till aktuell studie kommer att rekryteras från psykologstudentmottagningen vid Linköpings universitet, dit personer med mild till måttlig psykisk ohälsa söker sig för psykologisk behandling. I ett vidare perspektiv är det denna grupp som utgör den vanligaste patientgruppen i primärvården, vilket gör att generalisering till patienter i primärvård är möjlig.

Studien kommer att annonseras på internet och i dagspress. Efter att ha tagit del av information på vår hemsida kan försökspersonen anmäla sitt intresse och fylla i ett screeningformulär via krypterade formulär på internet. I samband med screening ger den tilltänkte deltagaren sitt godkännande enligt personuppgiftslagen (PUL) via internet, vilken också lämnas skriftligt vid inledande behandlingskontakt. Ambitionen är att ha så låga inklusionskrav som möjligt för att efterlikna en klinisk verklighet. Inklusionskriterierna är således utformade så att alla med någon form av depressionproblematik, som anses kunna bli hjälpta av behandlingens innehåll, skall inkluderas. En totalpoäng över 5 på Patient Health Questionnaire (PHQ-9) indikerar minimala depressionssymptom.

Innan slutlig inkludering genomgår den tilltänkta deltagaren en screening i form av den semistrukturerade kliniska intervjun Mini Internationell Neuropsykiatrisk Intervju (MINI) v.6 via telefonkontakt. Patienten besvarar följande självskattningsintrument: Beck Anxiety Inventory (BAl), Quality Of Life Inventory (QOLI), Alcohol Use Disorder Identification Test (AUDIT), Patient Health Questionnaire (PHQ-9) samt Beck Depression Inventory (BDI-II). En legitimerad specialistläkare i psykiatri kommer sedan att gå igenom resultaten från såväl screeningen som den strukturerade intervjun. Detta är särskilt viktigt då medicinsk behandling kan förekomma. Deltagarna i aktuell studie skall vara myndiga, ej samtidigt genomgå annan psykologisk behandling, ej bedömdas vara suicidala enligt MINI, inte ha ett pågående beroende, inte lida av allvarlig psykisk störning samt ha tillgång till internet och en smartphone. Detta är i linje med våra tidigare studier. Personer som exkluderas kommer att hänvisas till annan lämplig åtgärd. Personer som inte är intresserade av deltagande i studien erbjuds istället behandling enligt psykologstudentmottagningens rådande rutiner.

Behandlingsalliansen mäts före, under och efter behandling med självskattningsformuläret Working Alliance Inventory (WAI). Dessutom kommer data kring vårdkonsumtion och sjukfrånvaro att samlas in för en hälsoekonomisk analys. 12 och 24 månader efter avslutad behandling planeras uppföljningsmätningar göras i syfte att mäta behandlingens utfall över tid, för dessa administreras ovan nämnda självskattningsformulär.

Inkluderade försöksdeltagare kommer att randomiseras till någon av de två betingelserna: i) fyra live-terapi sessioner samt smartphone-applikation, som ett komplement och stöd till de fyra sessionerna, eller ii) 10 sessioner live-terapi, full beteendeaktivering. Smartphone-applikationen är ett program på mobiltelefonen, som dels använder sig av lösenordsskydd, dels av ett PIN-kod system. I programmet på mobiltelefonen förväntas deltagarna utföra uppgifter varje vecka som automatiskt rapporteras till behandlaren via krypterad överföring av datan. Behandlarna ger försöksdeltagarna återkoppling på framstegen och vägleder deltagarna i arbetet med att utföra aktiviteter som är viktiga i strävan mot att minska depressionssymptomen. Behandlarna är psykologkandidater på sista terminen av utbildningen och handleds av en erfaren psykoterapeut. Knuten till projektet är också en läkare som finns till hands då problem skulle uppstå (t ex om en deltagare skulle må sämre). All korrespondens mellan behandlaren och deltagaren kommer att arkiveras i enlighet med patientjournallagens riktlinjer. All hantering av data och inloggning hanteras enligt tidigare modeller som godkänts för system med internetförmedlad behandling.

*Tidsplan och utvärdering*

1. November 2012: Behandlingsprogram utformas och modifieras. Behandlare utbildas.
2. Januari 2013: Annons med länk till hemsidan. Anmälan till studien. Sökande skriver på samtycke enligt personuppgiftslagen (PUL). Screeningformulär fylls i.
3. Januari 2013: Första urvalet. Telefonintervjuer sker. Remisskonferens med läkare och forskningsledare. Randomisering. Besked till personer som exkluderats eller randomiseras till antingen behandlingsgrupp eller kontrollgruppen. Baslinjemått.
4. Januari - Mars 2013: Behandling.
5. Mars 2013: Slutmått. Uppföljningsintervjuer. Sammanställning av data.
6. Mars 2013: Kontrollgruppen erbjuds behandling
7. Höst 2013: Uppföljningsmätningar av den första behandlingsgruppen beräknas ske.

*Projektgrupp*

Gerhard Andersson, huvudansvarig för studien, Professor, leg. Psykolog, leg. Psykoterapeut,

Institutionen för Beteendevetenskap och Lärande, IBL, Linköpings universitet, LiU

Kien Hoa Ly, Doktorand, IBL, LiU

Christian Rück, Rück, specialistläkare psykiatri, Stockholm

Thomas Eriksson, specialistläkare psykiatri, Linköping

Naira Topooco Hjalmarsson, psykologkandidat termin 9, Karolinska institutet

*Referenser*

Andersson, G., & Cuijpers, P. (2009). Internet-based and other computerized psychological treatments for adult depression: A meta-analysis. *Cognitive Behaviour Therapy*, *38*, 196-205.

Andrews, G., Issakidis, C., Sanderson, K., Corry, J., & Lapsley, H. (2004). Utilising survey data to inform public policy: comparison of the cost-effectiveness of treatment of ten mental disorders. *The* *British Journal of Psychiatry,* Jun; 184:526-533.

Andrews, G., Cuijpers, P., Craske, M. G., McEvoy, P. & Titov, N. (2010). Computer therapy for the anxiety and depressive disorders is effective, acceptable and practical health care: A meta-analysis, *PLoS ONE 5.*

Boschen, M. J., & Casey, L. M. (2008). The use of mobile telephones as adjuncts to cognitive behavioral psychotherapy. *Professional Psychology: Research and Practice. 39*: p. 546-552.

Dimidjian, S., Barrera, M., Martell, C., Muñoz R. F. & Lewinsohn, P. M. (2011). The origins and current status of behavioral activation treatments for depression. *Annu Rev Clin Psych, 7*, 1–38

Glück, T. M., & Maercker, A. (2011). A randomized controlled pilot study of a breif web-based mindfulness training. *BMC Psychiatry, 11,* 1-12.

Harrison, V., Proudfoot, J., Wee, P. P., Parker, G., Pavlovic, D. H., & Manicavasagar, V. (2011). Mobile mental health: Review of the emerging field and proof of concept study. *Journal of Mental Health, 20,* 509-524.

Helbig, S., & Fehm, L. (2004). Problems with homework in CBT: rare exception or rather frequent? *Behavioural and Cognitive Psychotherapy, 32*, 291–301

Ly, K. H., Carlbring, P., & Andersson, G. (2012). Behavioral activation-based guided self-help treatment administered through a smartphone application: study protocol for a randomized controlled trial. *Trials.*13:62.

Ly, K. H., Dahl, J., Carlbring, P., & Andersson, G. (2012). Development and initial evaluation of a smartphone application based on acceptance and commitment therapy. *SpringerPlus*. 1:11.

Morris, M. E., Kathawala, Q., Leen, T. K., Gorenstein, E. E., Guilak, F., Labhard, M., et al. (2010). Mobile therapy: Case study evaluations of a cell phone application for emotional self-awareness. *Journal of Medical Internet Research, 12*, e10.

Newman, M. G., Szkodny, L. E., Llera, S. J. & Przeworski, A. (2011). A review of technology-assisted self-help and minimal contact therapies for anxiety and depression: is human contact necessary for therapeutic efficacy? *Clinical Psychology Review, 31,* 89-103.

Proudfoot, J., Klein, B., Andersson, G., Carlbring, P., Kyrios, M., et al. (2010). Guided CBT internet interventions: Specific issues in supporting clients with depression, anxiety and co-morbid conditions. In J. Bennett-Levy et al. (Eds.). *The Oxford Guide to low intensity CBT interventions* (pp. 253-264). Oxford: Oxford University Press.

SBU. (2007). *Datorbaserad kognitiv beteendeterapi vid ångestsyndrom eller depression*. Stockholm: Statens beredning för medicinsk utvärdering (SBU).

Sobocki. P. (2006). *Health economics of depression* (avhandling). Stockholm: Karolinska Institutet. Department of Learning, Informatics, Management and Ethics. Medical Management Centre.

Sobocki, P., Jönsson, B., Angst, J., & Rehnberg, C. (2006). Cost of depression in Europe. *Journal of Mental Health Policy and Economic, 9*, 87-98.

Sobocki, P., Lekander, I., Borgström, F., Ström, O. & Runeson, B. (2007). The economic burden of depression in Sweden from 1997 to 2005. *European Psychiatry*, *22*, 146-152

Socialstyrelsen. (2010). *Nationella riktlinjer för vård vid depression och ångestsyndrom – stöd för styrning och ledning*. Stockholm: Socialstyrelsen.
