# Supplementary material for: Smartphone-Supported versus Full Behavioural Activation for Depression: A Randomised Controlled Trial
Source: PLoS One. 2015 May 26;10(5):e0126559. doi: 10.1371/journal.pone.0126559 (PMC4444307; doi:10.1371/journal.pone.0126559)
Supplement: S3 Protocol — (DOC) [file pone.0126559.s007.doc]

# ANSÖKAN OM ETIKPRÖVNING

# Information till ansökan, *se bilaga och Vägledningar (*[*www.epn.se*](http://www.epn.se/)*)*

**Till Regionala etikprövningsnämnden i:** Linköping

Den regionala etikprövningsnämnd till vars upptagningsområde forskningshuvudmannen hör, se respektive nämnd *(*[*www.epn.se*](http://www.epn.se/)*)*

Avgift inbetald datum:

Observera att en ansökan aldrig är komplett och därmed kan behandlas förrän blanketten är korrekt ifylld och avgiften är betald.

**Projekttitel:** KBT-behandling med smartphone-stöd för behandling av depression.

Ange en beskrivande titel på svenska för lekmän, utan sekretesskyddad information. Ange också i förekommande fall projektets identitet, projektets/forskningsplanens (protokollets eller prövningsplanens) nummer, version, datum osv.

Projektnummer/identitet:       Version nummer:

## **Uppgifter som fylls i av den regionala etikprövningsnämnden**

Ansökan komplett: Dnr:

Begäran om ytterligare information (i sak): Begärd information inkommen:

Beslutsdatum: Expeditionsdatum:

**Ansökan avser (gäller även vid begäran om rådgivande yttrande):**

Forskning där endast en forskningshuvudman deltar (5 000 kr)

Forskning där mer än en huvudman deltar (16 000 kr)

Forskning där mer än en forskningshuvudman deltar, men där samtliga

forskningspersoner eller forskningsobjekt har ett omedelbart

samband med endast en av forskningshuvudmännen (5 000 kr)

Endast behandling av personuppgifter (5 000 kr)

Forskning som gäller klinisk läkemedelsprövning (16 000 kr)

Ändring av tidigare godkänd ansökan enligt 4 § förordning (2003:615) om

etikprövning av forskning som avser människor (2 000 kr)

Om nämnden finner att forskningsprojektet inte faller inom etikprövninglagens tillämpningsområde

önskas ett rådgivande yttrande. [(Info: 4a och 4b §§ i förordning 2003:615)](http://www.epn.se/media/8604/2003_615_4ab.doc) [(Info: Bilaga till ansökan)](http://www.epn.se/media/8525/bilaga.doc)

Ja:  Nej:

**1. Information om forskningshuvudman m.m.**

**1:1 Forskningshuvudman** ([Info: p. 1:1 i Vägledning till ansökan](http://www.epn.se/media/8601/vta_p1_1.doc))

Ansökan om etikprövning av forskning ska göras av forskningshuvudmannen. *Med forskningshuvudman avses en statlig myndighet eller en fysisk eller juridisk person i vars verksamhet forskningen utförs.* Inom staten utförs forskning främst vid lärosätena, men även vid vissa andra myndigheter, som t.ex. Brottsförebyggande rådet och Socialstyrelsen. Kommuner och landsting kan vara forskningshuvudmän, liksom privaträttsliga juridiska personer.

Namn: Linköpings universitet

Adress: IBL, 581 83 Linköping

**1:2 Behörig företrädare för forskningshuvudmannen**

Behörig företrädare är t.ex. prefekt, enhetschef, verksamhetschef. Forskningshuvudmännen bestämmer själva, genom interna arbets- och delegationsordningar eller genom fullmakt, vem som är behörig att företräda forskningshuvudmannen. Kopia av sådan handling *ska* bifogas.

Namn: Stefan Samuelsson Tjänstetitel: Prefekt

Adress: IBL, Linköpings universitet, 581 83 Linköping

**1:3 Forskare som är huvudansvarig för genomförandet av projektet (kontaktperson)**([Info: p. 1:3 i Vägledning till ansökan](http://www.epn.se/media/8714/vta_p1_3.doc))

Namn: Gerhard Andersson Tjänstetitel: Professor

Adress: IBL, Linköpings universitet

E-postadress: gerhard.andersson@liu.se

Telefon: 013-285840

Mobiltelefon: 0709-465257

**1:4 Plats** ([Info: p. 1:4 i Vägledning till ansökan](http://www.epn.se/media/8717/vta_p1_4.doc))

Plats (er) där projektet ska genomföras, ange inrättning (ar), institution (er), klinik (er) etc.

Institutionen för Beteendevetenskap och Lärande, Linköpings Universitet.

**1:5 Andra medverkande**

Övriga deltagande forskningshuvudmän samt forskare ansvariga för att lokalt genomföra projektet (kontaktpersoner) anges här eller i bilaga med namn och adresser (se p. 9 bilaga nr 1).

Se bilaga 1

**1:6 Ansökan/anmälan till andra myndigheter**

**Vid läkemedelsprövning**

Ansökan om tillstånd av *Läkemedelsverket* – se Läkemedelsverkets hemsida ([www.mpa.se](http://www.mpa.se/) )

Ansökan inlämnad (datum)       Tillstånd erhållits

EudraCT nr:

**Vid viss genetisk forskning**

Om personuppgifter om genetiska anlag som har framkommit efter genetisk undersökning kommer att hanteras i studien ska detta anmälas till *Datainspektionen* enligt 10 § personuppgiftsförordningen (1998:1191) – se Datainspektionens hemsida [www.datainspektionen.se](http://www.datainspektionen.se/lagar-och-regler/personuppgiftslagen/forhandskontroll/)

Anmälan inlämnad (datum)       Kommer att inlämnas efter godkänd etikprövning

**Vid viss forskning som innefattar bestrålning av forskningspersoner** ([Info: p. 9 i Vägledning till ansökan](http://www.epn.se/media/8747/vta_p9.doc))

Ansökan, enligt 16 och 22 §§ Strålsäkerhetsmyndighetens föreskrifter (SSMFS 2008:35) om allmänna skyldigheter vid medicinsk och odontologisk verksamhet med joniserande strålning, till *Strålskyddskommitté* –
för vidare information kontakta aktuell lokal strålskyddskommitté.

Ansökan inlämnad (datum):       Ansökan tillstyrkt

### 2. Uppgifter om projektet

**2:1 Sammanfattande beskrivning av forskningsprojektet (programmet)**[Vägledning till forskningsplan/forskningsprotokoll (program)](http://www.epn.se/media/8929/vägledning till forskningsplan.pdf) ([Info: p. 9 i Vägledning till ansökan](http://www.epn.se/media/8747/vta_p9.doc))

Beskrivningen ska kunna förstås av nämndens samtliga ledamöter. Undvik därför terminologi som kräver specialkunskaper. Ange bakgrund och syfte för studien samt den/de vetenskapliga frågeställning (ar) som man söker svar på. Ange de viktigaste undersökningsvariablerna. Beskriv vilka kunskapsvinster projektet kan förväntas ge och betydelsen av dessa. Ange om det är en registerstudie, uppdragsforskning etc. För fackmän avsedd detaljerad information i forskningsplan/forskningsprotokoll (program) *ska* bifogas som bilaga (se p. 9 bilaga nr 2). En utförligare beskrivning av studiens genomförande *avsedd för lekmän* kan vid behov bifogas den för fackmän avsedda obligatoriska forskningsplanen.

Vårdbehovet för psykologisk behandling för personer med depression är större än vad resurserna i hälso- och sjukvården idag kan erbjuda (Socialstyrelsen, 2010). År 2005 led 21 miljoner personer av depression i Europa och det kostar det europeiska samhället över 1000 miljarder SEK årligen (Sobocki, Jönsson, Angst & Rehnberg, 2006). Depression medför samhällskostnader i form av nedsatt produktivitet, ökad sjuklighet, ökad dödlighet samt ökad konsumtion av hälso- och sjukvård (Socialstyrelsen, 2010). I Sverige svarar depression och andra psykiska sjukdomar för en femtedel av den samlade sjukdomsbördan (Sobocki, 2006). Med sjukdomsbörda menas en kombination av antal förlorade friska år genom funktionsnedsättning och antalet förlorade år genom en för tidig död. Den totala kostnaden för depression i Sverige har fördubblats mellan år 1997 och 2005 och år 2005 kostade sjukdomen 32,9 miljarder kronor årligen (Sobocki, Lekander, Borgström, Ström & Runeson, 2007). Siffrorna beräknas både utifrån direkta kostnader såsom vårdkonsumtion och indirekta kostnader i form av sjukskrivningar och därmed förlorat produktionsvärde. Sannolikt är dessa siffror högre idag, då psykisk ohälsa anses vara ett växande problem. Ett exempel på denna utveckling är det ökade procentuella antalet personer som går långtidssjukskrivna eller har aktivitetsbidrag/förtidspensioneras (SBU, 2007).

För att behandla depression har kognitiv beteendeterapi (KBT) visat sig vara verksamt. I SBU:s samt Socialstyrelsens rekommendationer för behandling av depression hos vuxna, framhålls att effekten av KBT-behandling har starkt vetenskapligt underlag. Även psykofarmaka har visat på god effekt vid mätning direkt efter avslutad behandling. Dock är effekterna av psykofarmaka ofta kortvariga medan behandlingseffekterna av KBT är mer bestående och därmed har en förlängd skyddande effekt som läkemedelsbehandling saknar. Då läkemedel har en rad kända biverkningar, samt att långtidseffekten av de moderna antidepressiva läkemedlen ännu inte är helt kända, är KBT ett bra behandlingsalternativ. Detta motiveras vidare av Socialstyrelsens riktlinjer gällande KBT som förstahandsval vid behandling av mild till måttlig depression.

Även om traditionell KBT generellt har mycket gott stöd på gruppnivå så finns det patienter som inte fullföljer behandling och/eller inte rapporterar symtomlindring. Det finns ett stort antal patienter som avbryter behandlingen i förtid och följsamhet till obligatoriska hemuppgifter är ett stort problem (Helbig & Fehm, 2004). Dessa faktorer reducerar ofta behandlingsframgång och ökar risken för återfall. Således finns ett utvecklingsbehov gällande traditionell KBT-behandling. Man räknar t ex med att nuvarande metoder endast klarar av att minska sjukdomsbördan av depressiva sjukdomar med omkring en tredjedel (Andrews et al., 2004).

För att öka tillgängligheten av psykologisk behandling har en utveckling gått mot mer kostnads- och tidseffektiva behandlingsformat vid sidan av traditionell live-terapi (Newman, Szkodny, Llera & Przeworski, 2011). Förutom självhjälpslitteratur, även kallad biblioterapi, har en stor utveckling skett mot digitalt administrerad terapi, som t ex internetadministrerade behandlingar och behandling via Smartphones. Under de senaste 10 åren har flertalet datoradministrerade psykologiska behandlingar utvecklats för bland annat depression, stress, bipolär sjukdom, missbruksproblematik och ätstörningar (Harrison et al., 2011). Metaanalyser visar att internetadministrerade KBT-behandling ger måttliga till stora effekter på depressionssymptom (Andrews et al., 2010; Andersson & Cuijipers, 2009; Glück & Maercker, 2011). Forskningsfältet kring mobiltelefoni och psykisk hälsa är nytt, men flertalet studier pågår för att utveckla området (Proudfoot et al., 2010). Morris et al. (2010) sammanfattar att studierna är lovande och visar på möjligheten att leverera psykoterapi på ett nytt och effektivt sätt.

Stor erfarenhet av internetadministrerade KBT-behandlingar finns i forskargruppen. Nyligen gjordes också en stor randomiserad kontrollerad studie med en kombination av internetadministrerad behandling och smartphone-stöd för behandling av depression i forskargruppen. Denna studie blev tidigare godkänd av etikprövningsnämnden, dnr 2011/395-31. Resultaten visade på god effekt av internetadministrerad behandling med smartphone-stöd för behandling av depression.

Mot bakgrund av det goda forskningsstödet för traditionell KBT och digitalt administrerad KBT vid depression dras slutsatsen att en kombination av dessa behandlingar bör undersökas. Traditionell live-terapi vid depression, särskilt beteendeaktivering, antas kunna dra stor nytta av modern kommunikationsteknik, framför allt mobilteknik, för att göra behandlingen mer effektiv och till en integrerad del i klientens vardag. Beteendeaktivering som en behandlingsform för depression utgår från schemaläggning och hemuppgifter av aktiviteter. Ett särskilt program på mobiltelefonen, så kallad applikation, som fyller denna funktion förväntas kunna vara ett stöd i traditionell live-terapi som gör denna behandling mer effektiv. Denna applikation användes och testades i ovan nämnda studie.

Syftet med studien är att är att undersöka om live-terapi tillsammans med en smartphone-applikation, inriktad på att ge ett stöd i hemuppgifter samt en ökning av beteendeaktivering, är effektiv i behandling av mild till måttlig depression. Studien kommer att genomföras som en randomiserad kontrollerad behandlingsstudie där effekten av behandlingen jämförs med en kontrollgrupp. Eftersom det har visat sig att fullständig beteendeaktivering är en effektiv behandling av mild till måttlig depression (Dimidjian et al., 2011), kommer studien utformas som en så kallad non-inferiority-studie där behandlingsgruppen ges färre möten live, men istället stöd i form av smartphone-applikationen. Istället för en traditionell beteendeaktiverings-behandling på 10 sessioner, kommer behandlingsgruppen att ges fyra live-terapi sessioner samt smartphone-applikationen, som ett komplement och stöd till de fyra sessionerna. Som kontrollgrupp kommer vi att ge full beteendeaktivering med 10 sessioner, i live-terapi.

Om behandlingen skulle visa sig ha lika god effekt som kontrollgruppen skulle detta innebära ett starkt komplement till traditionell psykologisk behandling och psykofarmakologisk vård som erbjuds patienter med mild till måttlig depression. Om det visar sig att tekniskt stöd påverkar psykologisk behandling positivt så uppstår en mängd utvecklingsmöjligheter. Det kan på sikt leda till positiva konsekvenser såsom ökad tillgänglighet, minskade vårdköer samt effektivare behandling avseende kostnader och behandlingsutfall.

Deltagarna till aktuell studie kommer att rekryteras från psykologstudentmottagningen vid Linköpings universitet, dit personer med mild till måttlig psykisk ohälsa söker sig för psykologisk behandling. I ett vidare perspektiv är det denna grupp som utgör den vanligaste patientgruppen i primärvården, vilket gör att generalisering till patienter i primärvård är möjlig.

**2:2 Vilken/vilka vetenskaplig (a) frågeställning (ar) ligger till grund för projektets utformning?**

Om projektet kan karakteriseras som en hypotesprövning, ange den primära och eventuellt sekundära hypotesen. Hänvisning till mer detaljerad information för fackmän kan ske till bifogad forskningsplan enligt punkt 2:1

Frågeställningen som studien förväntas svara på är om live-terapi tillsammans med en smartphone-applikation, inriktad på att ge ett stöd i hemuppgifter samt en ökning av beteendeaktivering, är effektiv i behandling av mild till måttlig depression. Närmare specificerat:

Finns det några skillnader i hur deltagares depression påverkas, mätt genom självskattningar och klinisk intervju, mellan behandlingsgrupp och aktiv kontrollgrupp.

**2:3 Redogör för resultat från relevanta djurförsök**

Om djurförsök inte utförts ange skälen till detta.

Ej tillämpligt.

**2:4 Redogör översiktligt för undersökningsprocedur, datainsamling och datas karaktär**

([Info: p. 2:4 i Vägledning till ansökan](http://www.epn.se/media/8720/vta_p2_4.doc))

Av beskrivningen ska framgå hur projektet planeras genomföras. Beskriv insamlade datas karaktär. Ange hur datas tillförlitlighet säkerställs (t.ex. kvalitetskontroll/monitorering). - Vid enkäter och intervjuer ska beskrivas tillvägagångssätt och t.ex. frågors innehåll och hur slutsatser dras. Enkäter och skattningsskalor *ska* bifogas (se p. 9 bilaga nr 5). - För medicinsk forskning ska anges t.ex. typer av ingrepp, mätmetoder, antal besök, tidsåtgång vid varje försök, doser och administrationssätt för eventuella läkemedel och/eller isotoper, blodprovsmängd (även ackumulerad mängd vid multipla försök). Ange även om och på vilket sätt undersökningsprocedur m.m. skiljer sig från klinisk rutin. Ange proceduren för att ge den eventuella behandling efter projektets slut, som kan erfordras. Ange procedur för insamling av biologiskt material. Redogör för datakällor och procedurer vid behandling av personuppgifter. För mer detaljerad information kan hänvisning ske till bilagd forskningsplan.

Deltagarna till aktuell studie kommer att rekryteras från psykologstudentmottagningen vid Linköpings universitet, dit personer med mild till måttlig psykisk ohälsa söker sig för psykologisk behandling. I ett vidare perspektiv är det denna grupp som utgör den vanligaste patientgruppen i primärvården, vilket gör att generalisering till patienter i primärvård är möjlig.

Studien kommer att annonseras på internet och i dagspress (se Bilaga 3). Efter att ha tagit del av information på vår hemsida (se Bilaga 4a) kan försökspersonen anmäla sitt intresse och fylla i ett screeningformulär (se Bilaga 5) via krypterade formulär på internet. I samband med screening ger den tilltänkte deltagaren sitt godkännande via internet (även inkluderat information om enligt personuppgiftslagen - PUL (se bilaga 4b)), vilken också lämnas skriftligt vid inledande behandlingskontakt. Ambitionen är att ha så låga inklusionskrav som möjligt för att efterlikna en klinisk verklighet. Inklusionskriterierna är således utformade så att alla med någon form av depressionproblematik, som anses kunna bli hjälpta av behandlingens innehåll, skall inkluderas. En totalpoäng över 5 på Patient Health Questionnaire (PHQ-9) indikerar minimala depressionssymptom.

Innan slutlig inkludering genomgår den tilltänkta deltagaren en screening i form av den semistrukturerade kliniska intervjun Mini Internationell Neuropsykiatrisk Intervju (MINI) v.6 via telefonkontakt. Patienten besvarar följande självskattningsintrument: Beck Anxiety Inventory (BAl), Quality Of Life Inventory (QOLI), Alcohol Use Disorder Identification Test (AUDIT), Patient Health Questionnaire (PHQ-9) samt Beck Depression Inventory (BDI-II). En legitimerad specialistläkare i psykiatri kommer sedan att gå igenom resultaten från såväl screeningen som den strukturerade intervjun. Detta är särskilt viktigt då medicinsk behandling kan förekomma. Deltagarna i aktuell studie skall vara myndiga, ej samtidigt genomgå annan psykologisk behandling, ej bedömdas vara suicidala enligt MINI, inte ha ett pågående beroende, inte lida av allvarlig psykisk störning samt ha tillgång till internet och en smartphone. Detta är i linje med våra tidigare studier. Personer som exkluderas kommer att hänvisas till annan lämplig åtgärd. Personer som inte är intresserade av deltagande i studien erbjuds istället behandling enligt psykologstudentmottagningens rådande rutiner.

Behandlingsalliansen mäts före, under och efter behandling med självskattningsformuläret Working Alliance Inventory (WAI). Dessutom kommer data kring vårdkonsumtion och sjukfrånvaro att samlas in för en hälsoekonomisk analys. 12 och 24 månader efter avslutad behandling planeras uppföljningsmätningar göras i syfte att mäta behandlingens utfall över tid, för dessa administreras ovan nämnda självskattningsformulär.

Inkluderade försöksdeltagare kommer att randomiseras till någon av de två betingelserna: i) fyra live-terapi sessioner samt smartphone-applikation, som ett komplement och stöd till de fyra sessionerna, eller ii) 10 sessioner live-terapi, i form av beteendeaktivering. Smartphone-applikationen är ett program på mobiltelefonen, som dels använder sig av lösenordsskydd, dels av ett PIN-kod system. I programmet på mobiltelefonen förväntas deltagarna utföra uppgifter varje vecka som automatiskt rapporteras till behandlaren via krypterad överföring av datan. Behandlarna ger försöksdeltagarna återkoppling på framstegen och vägleder deltagarna i arbetet med att utföra aktiviteter som är viktiga i strävan mot att minska depressionssymptomen. Behandlarna är psykologkandidater på sista terminen av utbildningen och handleds av en erfaren psykoterapeut. Knuten till projektet är också en läkare som finns till hands då problem skulle uppstå (t ex om en deltagare skulle må sämre). All korrespondens mellan behandlaren och deltagaren kommer att arkiveras i enlighet med patientjournallagens riktlinjer. All hantering av data och inloggning hanteras enligt tidigare modeller som godkänts för system med internetförmedlad behandling.

**2:5 Redogör för om insamlat biologiskt material kommer att förvaras i en biobank** ([Info: p. 2:5 i Vägledning till ansökan](http://www.epn.se/media/8723/vta_p2_5.doc))

*Med biobank avses biologiskt material från en eller flera människor som samlas och bevaras tills vidare eller för en bestämd tid och vars ursprung kan härledas till den eller de människor från vilka materialet härrör.*Redogör för var och hur prover som ska sparas förvaras, kodningsprocedurer och villkor för utlämnande av prover. Ange huvudman för biobanken. Observera att i förekommande fall ska anmälan av biobank ske till Socialstyrelsen enligt lagen (2002:297) om biobanker i hälso- och sjukvården m.m.

Ej tillämpligt.

**2:6 Redovisa tillgång till nödvändiga resurser under projektets genomförande**

Ange vem/vilka som har ansvaret (prefekt, verksamhetschef eller motsvarande) för forskningspersonernas säkerhet vid alla enheter/kliniker där forskningspersoner ska delta. Intyg från dessa ansvariga *ska* bifogas (se p. 9 bilaga nr 9). Av intyget ska framgå att erforderliga ekonomiska, strukturella och personella resurser finns tillgängliga för att garantera forskningspersonernas säkerhet.

Se Bilaga 9.

**2:7 Journalföring, registrering och hantering av data** ([Info: p. 2:7 i Vägledning till ansökan](http://www.epn.se/media/8750/vta_p2_7.doc))

Redogör för hur undersökningsprocedurer och eventuella ingrepp journalförs. Ange hur registrering och behandling av resultaten ska gå till. Om materialet ska kodas, ange proceduren, vem som förvarar kodlistor och vem eller vilka som har tillgång till dem, var och hur länge de förvaras samt om materialet kommer att anonymiseras eller förstöras. Ange om band- och videoinspelningar används. Redogör för vilken tillgänglighet datamaterialet har och hur det förvaras samt hur erforderligt sekretesskydd erhålls.

För att skydda deltagarnas identitet kommer uppgifterna att vara kodade. Alla uppgifter kommer att behandlas strikt konfidentiellt och i enlighet med personuppgiftslagen (PUL). Data från frågeformulären kommer att registreras i avidentifierad form. Inga individuella data är möjliga att spåra i de vetenskapliga rapporter som presenteras från denna studie. Deltagarens identitet kan inte spåras genom resultaten som lagras. Identiteten kan endast identifieras med en särskild studiekod. Kodlistan med vars hjälp personlig information kan kopplas till individ, kommer att förvaras i pappersformat inlåst hos projektledaren vid Linköpings universitet. Resultatet av analyserna utlämnas ej till någon annan part, om inte detta krävs med laglig grund. Även deltagarens familj, läkare, försäkringsbolag och arbetsgivare inkluderas i begreppet "någon annan part". Forskningsdeltagarna kommer att använda personliga inloggningsuppgifter som krypteras. Data som lagras i smartphone-applikationen kommer att vara lagrat på databas med säkrade och krypterade anslutningar. All hantering av data och inloggning hanteras enligt tidigare modeller som godkännts för system med internetförmedlad behandling.

**2:8 Redogör för tidigare erfarenheter (egna och/eller andras) av den använda
proceduren, tekniken eller behandlingen**

Särskilt angeläget är att redovisning av risker för komplikationer görs tydliga och i förekommande fall med angivande av relevanta publikationer. Vid nya behandlingar av patienter, t.ex. med läkemedel, bör anges hur många patienter (med aktuell eller annan åkomma) som tidigare erhållit föreslagen behandling, läkemedelsdosering (eller annan dosering) samt hur långa behandlingsperioder som studerats.

En del av behandlingsmaterialet kommer att baseras på ett material som använts vid en tidigare studie vid Linköpings Universitet (Dnr 2011/395-31), vilket då resulterade i god effekt.

I övrigt finns stor erfarenhet av liknande studier i forskargruppen. Kognitiv beteendeterapi har i randomiserade kontrollerade studier gett goda resultat depressionsproblematik. Effekterna står sig väl i långtidsuppföljningar. I forskargruppen finns även stor erfarenhet av internet som del i KBT-behandlingar.

**3. Uppgifter om forskningspersoner**

**3:1 Hur görs urvalet av forskningspersoner?** ([Info: p. 3:1 i Vägledning till ansökan](http://www.epn.se/media/8726/vta_p3_1.doc))

*Med forskningsperson avses en levande människa som forskningen avser.*Ange urvalskriterier (inklusion och exklusion). Redogör för på vilket sätt forskaren kommer i kontakt med/får kännedom om lämpliga forskningspersoner. Ange om rekrytering sker från egna/andras tidigare eller pågående studier. Om annonsering sker, *ska* annonsmaterialet insändas som bilaga (se p. 9 bilaga nr 3). Om t.ex. barn eller personer som tillfälligt eller permanent inte är kapabla att ge ett eget informerat samtycke ska ingå i projektet, ska detta särskilt motiveras. Om vissa grupper (t.ex. kvinnor, barn eller äldre) utesluts från deltagande i projektet ska detta särskilt motiveras.

Deltagaren får kännedom om studien via psykologstudentmottagningen, internet och dagspress. Efter att ha tagit del av information på hemsidan anmäler personen intresse för deltagande. För deltagande i studien krävs att personen är myndig och svenskspråkig. Övriga inkluderingskrav kommer att baseras på screeningformulär samt diagnostisk intervju (se punkt 2:4). Studien kommer att inkludera personer med mild till måttlig depressionsprobematik. Även personer med subkliniska besvär kommer att inkluderas. Däremot kommer allvarlig psykopatologi, som bipolär sjukdom och psykossjukdomar, att exkluderas. Även de som precis startat med medicinering eller som håller på att ändra sin medicinering kommer att exkluderas. De som exkluderas kontaktas per telefon. Personer som exkluderas kommer att hänvisas till annan lämplig åtgärd varför exkludering inte befaras uppfattas negativt. Personer som inte är intresserade av deltagande i studien erbjuds istället behandling enligt psykologstudentmottagningens rådande rutiner.

**3:2 Ange relationen mellan forskare/försöksledare och forskningspersonerna**

Behandlare (t.ex. läkare, psykolog, sjukgymnast) - forskningsperson (t.ex. patient, klient)

Kursgivare (lärare) - student

Arbetsgivare - anställd

Annan relation som kan tänkas medföra risk för påverkan. Beskriv:

3:3 Redogör för det statistiska underlaget för studiepopulationens (-ernas)/ undersökningsmaterialets (-ens) storlek [**(Info: p. 3:3 i Vägledning till ansökan)**](http://www.epn.se/media/8729/vta_p3_3.doc)

Redovisa statistisk styrka, så kallad ”power”- beräkning eller redovisa motsvarande överväganden som tydliggör studiens möjligheter att besvara frågeställningarna.

I studien kommer 50 personer att inkluderas i behandlingsgrupp och 50 till aktiv kontrollgrupp. Eftersom vi kommer att betrakta en effektstorlek på Cohen's d=0.50 (mean standardized difference) som en kliniskt signifikan skillnad är 50 personer i varje behandlingsarm fullt tillräckligt för att kunna visa på en effekt (givet 80% power).

**3:4 Ange om forskningspersonerna kan komma att inkluderas i flera studier samtidigt
eller i annan/andra studie (-er) i nära anslutning till denna? I så fall vilken typ av forskning?** ([Info: p. 3:4 i Vägledning till ansökan](http://www.epn.se/media/8732/vta_p3_4.doc))

Nej.

3:5 Vilket försäkringsskydd finns för de forskningspersoner som deltar i projektet?

Det åligger forskningshuvudmannen att kontrollera att befintliga försäkringar täcker eventuella skador som kan uppkomma.

Ej tillämpligt.

**3:6 Vilken ekonomisk ersättning eller andra förmåner utgår till de forskningspersoner
som deltar i projektet och när betalas ersättningen ut?** Utförligare beskrivning kan lämnas i bilaga. ([Info: p. 3:6 i Vägledning till ansökan](http://www.epn.se/media/8735/vta_p3_6.doc))

Ersättning för obehag och besvär. Belopp (före skatt):

Ersättning för förlorad arbetsinkomst  Ja  Nej

Reseersättning  Ja  Nej

Befrielse från kostnader för läkemedel  Ja  Nej

Befrielse från andra kostnader. Vilka?

Andra förmåner. Vilka?

När betalas ersättningen ut?

Ingen ersättning betalas ut

### 4. Information och samtycke ([**Info: Forskningspersonsinformation**](http://www.epn.se/media/8598/forskningspersonsinformation.doc))

4:1 Proceduren för och innehållet i den *information* som lämnas då forskningspersoner tillfrågas om deltagande

Beskriv hur och när information ges och vad den innehåller. Ange vem som informerar. Normalt ska en kortfattad och lättförståelig skriftlig information ges. Denna skriftliga information *ska* bifogas ansökan (se p. 9 bilaga nr 4). Om ingen eller ofullständig information ges, måste skälen för detta noggrant anges.

Fullständig information till forskningsdeltagarna ges innan screening telefonledes av behandlaren (se bilaga 4a) och via projektets hemsida. Denna information finns tillgänglig under studiens gång. Frågor kring studiens upplägg besvaras av forskningsledaren. Om medicinska frågor uppstår i denna fas besvaras dessa av projektets läkare.

**4:2 Hur och från vem inhämtas *samtycke*?**

Beskriv proceduren; vem som frågar, när detta sker och hur samtycket dokumenteras. Utförlig redovisning är särskilt viktig då barn eller personer med nedsatt beslutskompetens ingår i studien, likaså vid studier av en grupp/grupper, t.ex. föreningar, organisationer, företag, kyrkosamfund, församlingar eller skolklasser.

Information kommer att finnas tillgänglig på projektets hemsida. För att deltaga i studien måste samtycke ges. Samtyckesformulär finns tillgängligt på hemsidan (se bilaga 4b), underskrift ges vid första bedömningssamtalet i behandlingen. Försökspersonen behöver intyga samtycke genom att skriva in sitt personnummer vid två tillfällen. Se vidare information till försökspersoner (se bilaga 4a).

### 5. Forskningsetiska överväganden

5:1 Redogör för de risker som deltagandet kan medföra samt möjliga komplikationer

Dessa kan vara t.ex. fysisk skada, smärta, obehag eller integritetsintrång som projektet innebär eller kan innebära. Ange vilka åtgärder som har vidtagits för att förebygga de risker som nämns ovan samt vilken beredskap som finns för att hantera sådana komplikationer. Ange vilka/de metoder som kommer att användas för att efterforska, registrera och rapportera oönskade händelser.

Deltagandet i studien är frivilligt. Deltagarna är alltid fria att avbryta sitt deltagande om de så önskar. Om detta informeras forskningspersonerna innan behandlingen påbörjas (se Bilaga 4a). Ett visst integritetsintrång kan upplevas i alla psykologiska behandlingar, så även i denna. Vidare kan det finnas en oro för brister i datasäkerheten från deltagarnas sida. För att förebygga detta så kommer en tydlig beskrivning över hanteringen av personuppgifter att finnas tillgänglig via hemsidan som personerna tar del och av och godkänner innan de bestämmer sig för att delta.

Vid all psykologisk behandling är bedömningen av suicidrisk en viktig del. Deltagare som vid screening anses uppvisa tecken på suicidrisk kommer, som tidigare redovisats, exkluderas från studien och uppmanas att söka psykiatrisk vård på hemorten. Om suicidrisk bedöms kommer aktiv hjälp ges till deltagaren för att komma i kontakt med lämplig psykiatrisk vård på hemorten. Uppstår akut behov under studiens gång kommer behandlingsansvarig se till att lämpliga åtgärder vidtas. De personer som exkluderas kan komma att uppfatta detta negativt. För att förebygga detta kommer en tydlig beskrivning av exklusionskriterier redovisas på hemsidan. De individer som tackar nej till att delta i studien kommer att erbjudas psykologisk behandling vid psykologstudentmottagningen på sedvanligt sätt. De eventuella individer som efter studiens screening mot förmodan inte längre uppfyller studentmottagningens inklusionskriterier kommer att hänvisas till lämplig åtgärd.

Det är viktigt att forskningsdeltagaren känner trygghet med Smartphone-applikationen och att andra alternativ ställs till förfogande om så ej blir fallet. Deltagarna kommer att informeras om hur de skyddar sina mobiltelefoner mot intrång. Förutom skydd genom lösenord kommer deltagarna att få instruktioner att använda både telefonens och stödprogrammets PIN-kod system. Utifrån rådande KBT-principer är forskningsdeltagarens kontinuerliga utvärdering och delaktighet en självklar komponent i behandlingen.

5:2 Redogör för förutsebar nytta för de forskningspersoner som ingår i projektet

Förväntningarna på studien består i att merparten av deltagarna som genomgått behandling kommer att uppleva reducerade besvär med psykiska besvär i allmänhet och besvär relaterat till depression i synnerhet. En viss delmängd deltagare förväntas även uppnå en sådan förbättring att de kan sägas vara i remission från den depressionsdiagnos de tidigare uppfyllt.

Deltagarna erbjuds en kostnadsfri behandling utifrån beprövad erfarenhet och enligt evidensbaserade psykologiska metoder. Det är troligt att flertalet deltagare kan uppnå en förbättring. Detta baseras på att de använda KBT-metoderna i tidigare studier visat sig verksamma, vilket framgår av SBU-rapporten "Behandling av depressionssjukdomar".

5:3 Gör en egen värdering av förhållandet risk - nytta för de forskningspersoner som deltar

Den huvudsakliga risken består i att försökspersonerna lägger ned tid och kraft på en behandling som inte genererar positiva resultat. Forskningsgruppen bedömer dock att behandlingen bör kunna reducera depression och i viss mån ångestbesvär, samt att livskvaliteten hos merparten av deltagarna i behandlingsgruppen bör förbättras.

Eventuella problem och missnöjesyttringar som uppstår under studiens gång kommer hanteras i samråd med studieansvarig och läkare. Nyttan överskrider enligt vår bedömning de eventuella risker som deltagandet kan medföra. Riskbedömningen för denna typ av behandling bedömer vi som likvärdig med traditionell KBT-behandling.

**5:4 Identifiera och precisera om etiska problem t.ex risk - nytta i ett vidare perspektiv
kan uppstå inom eller genom projektet**

Här kan redovisas om exempelvis vissa grupper kan komma att utpekas/få hjälp som ett resultat av studien.

En eventuell risk med denna Smartphone-stödda KBT-behandling är att den får en exkluderande funktion för personer som inte känner sig trygga med att hantera Smartphones. Dock bör behandlare alltid ta hänsyn till klientens önskemål om behandlingens utformning och förutsättningar. Hur behandlingen präglas av Smartphone-applikationen är flexibelt och valbart utifrån både behandlare och forskningsdeltagares önskemål. Således minimeras risken att forskningsdeltagaren uppfattar att det krävs en hög teknisk kompetens för att få hjälp. Vidare har Smartphone-applikationen redan testats i en randomiserad kontrollerad studie. Inga specifika negativa yttringar om tekniska svårigheter har framkommit.

Nyttan med denna studie är den effekt som kan komma att upplevas direkt av den behandlade patienten. Vidare att mångfalden av vårdmöjligheter ökar tack vare den nya kombinationen av traditionell KBT-behandling och digitalt administrerad KBT-behandling.

Då det finns ett stort antal personer som inte blir hjälpta av KBT-behandling idag finns incitament för att förbättra och effektivisera behandling. Denna Smartphone-stödda behandling är ett försök till detta. Studier på internetbehandling har visat goda resultat trots att behandlare och patient aldrig möts i samma fysiska rum. Slutsatsen dras att Smartphone-applikationen kan användas för att upprätthålla kontinuitet i behandling utan att den blir avhänging att behandling sker i ett fysiskt rum. I ett samhällsperspektiv bör också påpekas att det i Sverige finns färre terapeuter med erforderlig kompetens att ge psykologisk behandling än vad det finns människor som är i behov av denna vård. Vidare saknar vissa personer praktisk eller ekonomisk möjlighet att få ta del av psykologisk behandling. Nyttan med denna studie kan därför vara att valmöjligheten och tillgängligheten för personer i behov av behandling för depression ökar.

Det tekniska stödet främjar personens möjlighet att vidmakthålla sitt uppnådda behandlingsresultat tack vare tillgänglighet till organiserade beteendeaktiverande aktiviteter. Om klienten får bakslag efter avslutad behandling kan denne på egen hand repetera behandlingen vilket kan minska risken för kraftigt återfall. Detta kan innebära positiva konsekvenser för samhället i form av minskad vårdbelastning och antal sjukskrivningar.

### 6. Redovisning av resultaten

**6:1 Hur garanteras forskningshuvudmannen och medverkande forskare tillgång till data (anges vid t.ex. uppdragsforskning) och vem ansvarar för databearbetning och rapportskrivning?**([Info: p. 6:1 i Vägledning till ansökan](http://www.epn.se/media/8744/vta_p6_1.doc))

Professor Gerhard Andersson, leg psykolog och leg psykoterapeut och doktorand Kien Hoa Ly har huvudansvaret för databearbetningen och rapportskrivning.

6:2 Hur kommer resultaten att göras offentligt tillgängliga? Kommer studien att insändas för publicering i tidskrift eller publiceras på annat sätt?

Ange i vilken form resultaten planeras offentliggöras samt tidsplan för detta.

Resultaten kommer att publiceras i nationella och internationella vetenskapliga tidskrifter samt presenteras vid konferenser. En sammanfattning läggs ut på nätet för lekmän samt deltagare i studien.

6:3 På vilket sätt garanteras forskningspersonernas rätt till integritet när materialet offentliggörs/publiceras?

Redovisas resultat på statistisk gruppnivå? Beskriv procedurer eller metoder för avidentifiering/anonymisering.

Resultaten kommer huvudsakligen redovisas på gruppnivå och ingen enskild person, kommer att kunna identifieras i sammanställningen.

7. Redovisning av ekonomiska förhållanden och beroendeförhållanden

Redovisning enligt punkterna 7:1-7:3 syftar till att tydliggöra alla direkta eller indirekta förhållanden, som kan tänkas påverka forskarens relation till forskningspersonerna (vid t.ex. informations-, samtyckes-, genomförandeprocedurer).

7:1 Vid uppdragsforskning

Ange uppdragsgivaren t.ex. ett företag (vid klinisk läkemedelsprövning eller prövning av andra nya produkter), en organisation eller en myndighet.

Namn:       Kontaktperson:

Adress:       Telefon/mobiltelefon:

Ange uppdragsgivarens relation till forskningshuvudmannen/medverkande forskare, t.ex.
anställningsförhållande

Ej tillämpligt.

**7:2 Redovisa eventuella ekonomiska överenskommelser med uppdragsgivare eller**

**andra finansiärer (namn, belopp)**

Vid klinisk läkemedelsprövning bör hänvisning ske till ingånget avtal med sjukvårdshuvudmannen. Liknande överenskommelser kan förekomma vid annan uppdragsforskning och ska redovisas på samma sätt. Separata överenskommelser med den/de som ska genomföra forskningen ska redovisas. Belopp som kommer att erhållas för studien/ersättning till kliniken/genomföraren, vad ersättningen ska täcka och ev. belopp som erhålls per forskningsperson, ska också anges här (se p. 9 bilaga nr 12).

Ej tillämpligt.

7:3 Redovisa forskningshuvudmannens, huvudansvarig forskares och medverkande forskares egna intressen

Här redovisas t.ex. aktieinnehav, anställning, konsultuppdrag i finansierande företag, eget företag som kan få (direkt eller indirekt) ekonomisk vinst av forskningen (se p. 9 bilaga nr 12).

Ej tillämpligt.

**8. Undertecknande**

Behörig företrädare för sökande forskningshuvudman enligt p. 1:2

Ort: Linköping Datum: 2012-11-14

Signatur: __________________________________________________________________

Namnförtydligande: Stefan Samuelsson

Tjänstetitel: Prefekt

Undertecknad forskare som genomför projektet (kontaktperson) enligt p. 1:3 intygar härmed att forskningen kommer att genomföras i enlighet med ansökan

Ort: Linköping Datum: 2012-11-14

Signatur: _________________________________________________________________

Namnförtydligande: Gerhard Andersson

Tjänstetitel: Professor

**9. Förteckning över bilagor** ([Info: p. 9 i Vägledning till ansökan](http://www.epn.se/media/8747/vta_p9.doc))

Dokument som, i tillämpliga fall, ska bifogas *om inte motsvarande information finns i blanketten* har markerats med x. Markera de bilagor som skickas in med denna ansökan.

| **Insänd med ansökan** | **Bil nr** | Beskrivning | **Klinisk läkemedels-**  **prövning** | **Annan forskning** |
| --- | --- | --- | --- | --- |
|  | 1 | Deltagande forskningshuvudmän och medverkande forskare (kontaktpersoner) vid forskning där mer än en forskningshuvudman deltar. Info p. 1:5 | x | x |
|  | 2 | För fackmän avsedd forskningsplan, vid behov även för lekmän avsedd bilaga. Info p. 2:1 och i Vägledning till forskningsplan/forskningsprotokoll (program) | x | x |
|  | 3 | Annonsmaterial för rekrytering av forskningspersoner. Info p. 3:1 och i Vägledning till ansökan p. 3:1 | x | x |
|  | 4 | Skriftlig information till dem som tillfrågas. Info p. 4:1 och i Forskningspersonsinformation | x | x |
|  | 5 | Enkät, frågeformulär. Info p. 2:4 | x | x |
|  | 6 | Gemensam EU blankett (gäller fr.o.m. den 1 maj 2004), gäller även vid ändring. | x |  |
|  | 7 | Sammanfattning av protokollet på svenska | x |  |
|  | 8 | Prövarhandbok alt. bipacksedel/produktresumé/IB | x |  |
|  | 9 | Intyg från verksamhetschef/motsv. om resurser och om forskningspersonernas säkerhet. Info p. 2:6 | x | x |
|  | 10 | CV för forskare (samma som p. 1:3) med huvudansvar för genomförandet, redovisa forskarens (-arnas) kompetens av relevans för studien. Info i Vägledning till ansökan p. 1:3 | x | x |
|  | 11 | Beskrivning av ersättning till forskningspersoner. Info p. 3:6 och  i Vägledning till ansökan p. 3:6 | x | x |
|  | 12 | Överenskommelser med uppdragsgivare/finansiär om  t.ex. anställningsförhållanden, bidrag/ersättning till prövningsplats, sjukvårdshuvudman, forskningshuvudman eller forskare. Info p. 7:2 och p. 7:3 | x | x |

**Övriga bilagor som bifogas ansökan:**
